# Effect of Clinical Decision Support on Cardiovascular Risk Among Adults With Bipolar Disorder, Schizoaffective Disorder, or Schizophrenia: A Cluster Randomized Clinical Trial

**DOI:** 10.1001/jamanetworkopen.2022.0202

**Published:** 2022-03-07

**Authors:** Rebecca C. Rossom, A. Lauren Crain, Patrick J. O’Connor, Stephen C. Waring, Stephanie A. Hooker, Kris Ohnsorg, Allise Taran, Kristen M. Kopski, JoAnn M. Sperl-Hillen

**Affiliations:** 1 Department of Research, HealthPartners Institute, Minneapolis, Minnesota; 2 Essentia Health and Essentia Institute of Rural Health, Duluth, Minnesota; 3 Park Nicollet Health Services, Minneapolis, Minnesota; 4 Now with Medica Health Plan, Minnetonka, Minnesota

## Abstract

**Importance:**

Adults with schizophrenia, schizoaffective disorder, or bipolar disorder, collectively termed *serious mental illness* (SMI), have shortened life spans compared with people without SMI. The leading cause of death is cardiovascular (CV) disease.

**Objective:**

To assess whether a clinical decision support (CDS) system aimed at primary care clinicians improves CV health for adult primary care patients with SMI.

**Design, Setting, and Participants:**

In this cluster randomized clinical trial conducted from March 2, 2016, to September 19, 2018, restricted randomization assigned 76 primary care clinics in 3 Midwestern health care systems to receive or not receive a CDS system aimed at improving CV health among patients with SMI. Eligible clinics had at least 20 patients with SMI; clinicians and their adult patients with SMI with at least 1 modifiable CV risk factor not at the goal set by the American College of Cardiology/American Heart Association guidelines were included. Statistical analysis was conducted on an intention-to-treat basis from January 10, 2019, to December 29, 2021.

**Intervention:**

The CDS system assessed modifiable CV risk factors and provided personalized treatment recommendations to clinicians and patients.

**Main Outcomes and Measures:**

Patient-level change in total modifiable CV risk over 12 months, summed from individual modifiable risk factors (smoking, body mass index, low-density lipoprotein cholesterol level, systolic blood pressure, and hemoglobin A_1c_ level).

**Results:**

A total of 80 clinics were randomized; 4 clinics were excluded for having fewer than 20 eligible patients, leaving 42 intervention clinics and 34 control clinics. A total of 8937 patients with SMI (4922 women [55.1%]; mean [SD] age, 48.4 [13.5] years) were enrolled. There was a 4% lower rate of increase in total modifiable CV risk among intervention patients relative to control patients (relative rate ratio [RR], 0.96; 95% CI, 0.94-0.98). The intervention favored patients who were 18 to 29 years of age (RR, 0.89; 95% CI, 0.81-0.98) or 50 to 59 years of age (RR, 0.93; 95% CI, 0.90-0.96), Black (RR, 0.93; 95% CI, 0.88-0.98), or White (RR, 0.96; 95% CI, 0.94-0.98). Men (RR, 0.96; 95% CI, 0.94-0.99) and women (RR, 0.95; 95% CI, 0.92-0.97), as well as patients with any SMI subtype (bipolar disorder: RR, 0.96; 95% CI, 0.94-0.99; schizoaffective disorder: RR, 0.94; 95% CI, 0.90-0.98; schizophrenia: RR, 0.92; 95% CI, 0.85-0.99) also benefited from the intervention. Despite treatment effects favoring the intervention, there were no significant differences in individual modifiable risk factors.

**Conclusions and Relevance:**

This CDS intervention resulted in a rate of change in total modifiable CV risk that was 4% lower among intervention patients compared with control patients. Results were driven by the cumulative effects of incremental and mostly nonsignificant changes in individual modifiable risk factors. These findings emphasize the value of using CDS to prompt early primary care intervention for adults with SMI.

**Trial Registration:**

ClinicalTrials.gov Identifier: NCT02451670

## Key Points

**Question:**

Does a clinical decision support system aimed at primary care clinicians improve cardiovascular health for people with serious mental illness?

**Findings:**

In this cluster randomized clinical trial of 8937 patients with serious mental illness, intervention patients’ rate of change in total modifiable cardiovascular risk over 12 months was 4% lower than control patients’ rate of change. There were no significant differences in individual modifiable risk factors.

**Meaning:**

Although treatment effects favored the intervention, the results were driven by cumulative effects of incremental and mostly nonsignificant changes in individual modifiable cardiovascular risk factors.

## Teaser

This randomized clinical trial assesses whether a clinical decision support system aimed at primary care clinicians improves cardiovascular health for adult primary care patients with serious mental illness.

## Introduction

People with bipolar disorder, schizophrenia, or schizoaffective disorder, collectively termed *serious mental illness* (SMI), die at 2.3 times the rate of people without SMI, shortening their life spans by 10 to 15 years.^1,2^ Cardiovascular (CV) disease is the leading cause of death for people with SMI,^3^ associated in part with higher relative risks of dyslipidemia (5-fold), smoking (2-fold to 3-fold), diabetes (2-fold), and obesity (1.5-fold to 2-fold).^4,5^ Some SMI medications may increase cardiometabolic risk by adversely affecting weight, insulin resistance, and lipid metabolism.^6,7,8^ In theory, excess CV mortality among people with SMI could be reduced by early recognition and management of modifiable CV risk factors, among other strategies.^3,9,10,11^

Clinical decision support (CDS) tools are typically technology-based interventions that provide patient-specific information, often at the point of care, to improve health and health care.^12^ Clinical decision support in primary care settings could help prompt clinicians to address gaps in evidence-based care, but many previous CDS studies have had null results, in part owing to poor CDS design and low use of the CDS tools.^13,14,15,16,17,18^ With improved design and implementation, more recent studies of electronic health record (EHR)–linked CDS tools have achieved high use rates and decreased CV risk in populations without SMI.^19,20,21^ This pragmatic cluster randomized clinical trial, designed to test the effectiveness of the intervention in real-world clinical practice, assessed whether an EHR-linked CDS system slowed increases in modifiable CV risk among adults with SMI.

## Methods

### Study Design and Study Setting

The design and methods of this cluster randomized clinical trial have been previously described.^22^ The trial protocol and statistical analysis plan can be found in Supplement 1. This report follows the Consolidated Standards of Reporting Trials (CONSORT) reporting guideline for cluster randomized studies. The study was conducted from March 2, 2016, to September 19, 2018, at 76 primary care clinics in 3 health care systems providing integrated health care for patients in Minnesota, North Dakota, and Wisconsin: Essentia Health, HealthPartners, and Park Nicollet. Study procedures were reviewed and approved by each site’s institutional review board. Waivers of consent for clinicians and patients were granted by each site’s institutional review board because CDS guidance was limited to evidence-based recommendations from national guidelines.

### Clinic Randomization

Primary care clinics that treated at least 20 patients with SMI in the prior year were randomized by the study statistician (A.L.C.) using site-stratified restricted randomization to balance site-specific factors that could affect the intervention or its implementation.^22,23^ Site A clinics were balanced on the proportion of patients insured by Medicaid, the presence of onsite behavioral health services, and the number of patients with SMI. Site B clinics were balanced on urbanicity, the proportion of patients who smoked, and the proportion of patients younger than 30 years. Site C clinics were balanced on the proportion of patients insured by Medicaid, the proportion of patients receiving optimal vascular care, and the number of patients with SMI. Cluster randomization was chosen to minimize contamination. Given the nature of the intervention, clinicians were not blinded to clinic assignments.

### Study Participants

Participant enrollment began on March 2, 2016, at site A; October 18, 2016, at site B; and March 15, 2017, at site C. Enrollment ended on September 19, 2017, and patients were followed up through September 19, 2018. Adults aged 18 to 75 years who were not pregnant, had SMI, had at least 1 modifiable CV risk factor not at the goal set by the American College of Cardiology/American Heart Association (ACC/AHA) guidelines, and had an index visit and at least 1 postindex visit at any randomized clinic were eligible. Patients in nursing homes or hospice, requesting exclusion from research, or with active cancer diagnoses were excluded from analyses. An index visit was the first visit made by an eligible patient during the intervention. Patients were assigned to their index visit clinic’s treatment group. Patients were considered to have SMI if they had at least 1 inpatient or 2 outpatient EHR-documented diagnoses of bipolar disorder, schizophrenia, or schizoaffective disorder in the 2 years prior to the index visit (eTable 1 in Supplement 2). Patients with *International Classification of Diseases, Ninth Revision* and *International Statistical Classification of Diseases and Related Health Problems, Tenth Revision* codes crossing SMI subtypes were considered to have schizoaffective disorder.

### Intervention

For study-eligible patients, intervention clinic rooming staff members received EHR best practice advisories prompting them to print and distribute 1-page handouts for clinicians and patients. The clinician handout summarized and prioritized each patient’s modifiable CV risk factors: blood pressure, lipid levels, glucose and hemoglobin A_1c_ (HbA_1c_) levels, smoking status, and body mass index (BMI; calculated as weight in kilograms divided by height in meters squared). The clinician handout also estimated the patient’s 10-year (for those aged 40-75 years) and/or 30-year (for those aged 18-59 years) ACC/AHA CV risk and gave patient-specific treatment recommendations based on national guidelines (eg, ACC/AHA,^24,25^ Framingham Heart Study,^26^ and United Kingdom Prospective Diabetes Study^27,28^). The clinician handouts included patient-specific suggestions regarding medications, diet, exercise, and smoking cessation. The patient handouts contained similar information designed for individuals with lower literacy levels, including suggestions regarding diet, exercise, lifestyle programs, and smoking cessation. Handouts were designed to be used as shared decision-making tools, and rooming staff members suggested that patients review and talk to their clinicians about the handouts. There were no alerts or printouts in control clinics. For study-eligible patients who presented for a subsequent visit with a psychiatric prescriber, the CDS alerted the prescriber when patients had active prescriptions of potentially obesogenic SMI medications (eTable 2 in Supplement 2) and had elevated BMIs or recent weight gain.

### Data Collection

The CDS system ran in the background to collect EHR data, including demographic characteristics, vital signs, medications, comorbidities, allergies, and laboratory data, for study-eligible patients in intervention and control clinics for 2 years preceding the visit. Race and ethnicity were self-reported to health care systems and included Asian, Black, Hispanic, Native American, White, and other or unknown race and ethnicity (the “other or unknown race and ethnicity” group included people who were Pacific Islander and people for whom there were no race or ethnicity data recorded in the electronic health record). Electronic health record data were harvested to obtain safety event information, including suicidal ideation (as recorded on the Patient Health Questionnaire^29^), hospitalizations, and emergency department visits.

### Outcomes

The primary outcome was patient-level rate of change in total modifiable CV risk during the 12 months after the index visit. Secondary outcomes were rates of change in individual modifiable CV risk factors (blood pressure, lipid levels, HbA_1c_ level, smoking, BMI) and use of potentially obesogenic SMI medications in the 12 months after the index visit. Total modifiable CV risk and individual CV risk factors were calculated by the CDS at each clinic visit. Total modifiable CV risk was defined as the sum of modifiable CV risk factors, calculated via the following. (1) Ten-year risk equations estimated CV risk (modifiable plus nonmodifiable [age, sex, race and ethnicity]) using ACC/AHA^24,25^ and Framingham risk equations.^26^ For patients younger than 40 years, 10-year risk equations were calculated as if the patient were 40 years of age. (2) A risk component for each modifiable CV risk factor was calculated as the difference between the calculated risk using the patient’s values and the goal using ACC/AHA,^24,25^ Framingham Heart Study,^26^ and United Kingdom Prospective Diabetes Study equations.^27,28^ For BMI, the goal was a decrease of 3 BMI units for those with a BMI of 28 or more or a decrease to a BMI of 25 for those with BMIs of 25 to 27.9. Risk components for modifiable CV risk factors at the goal set by the ACC/AHA guidelines were calculated as zero. (3) Modifiable CV risk components were summed to calculate total modifiable CV risk.

### Statistical Analysis

Statistical analysis was conducted on an intention-to-treat basis from January 10, 2019, to December 29, 2021. Total modifiable CV risk and individual modifiable CV risk factors were analyzed using general or generalized linear mixed models with distribution-appropriate link functions (eg, log-binomial function and log-negative binomial function) and a random patient × clinic intercept. Outcomes measured repeatedly per patient (modifiable CV risk, blood pressure, lipid levels, HbA_1c_ level, BMI) were predicted from fixed effects of treatment, linear time in years from the index visit to each outcome, and the treatment × time interaction. The treatment effect was quantified as the rate of change between the index visit and 12 months after the index visit among patients in intervention clinics (rate ratio [RR]) relative to the RR among patients in control clinics (ie, RR for treatment × time). The 2-sided 95% CI around RR for treatment × time assessed its statistical significance. Covariates included sex, age, outcome value at the index visit, site, and balancing covariates.

The smoking status model estimated the likelihood that patients who were documented as current smokers at the index visit were documented as having quit at their last visit in the follow-up period. This model did not include a random patient intercept or baseline outcome value and added a covariate for the log-years elapsed between the index visit and the last visit. The obesogenic medication model also used this approach and was applied to patients with an active prescription for an obesogenic medication at the index visit and with either 7% or more weight gain in the previous 12 months or a most recent BMI increase greater than 2. Subgroup analyses by age and sex (a priori) and SMI subtype and race and ethnicity (post hoc) were conducted by adding subgroup main effect and interaction terms to these models. Confidence intervals around the subgroup treatment effects were not adjusted for multiple comparisons and should be considered exploratory. Study analyses were conducted using SAS, version 9.4 software (SAS Institute Inc).

### Sample Size

An a priori power analysis estimated the minimum detectable standardized effect for the time × treatment parameter in a mixed-effects regression model (power = 0.80; 2-sided α = .05) estimating a normally distributed outcome. Assumptions were 52 clinics randomized on a 1:1 basis and 2250 patients with SMI, equally distributed across clinics with 4 outcome measures each (interclass correlation coefficient = 0.01, 0.02 for clinics; interclass correlation coefficient = 0.35, 0.90 for patients). The resulting minimum detectable standardized effect (Cohen *d* < 0.10) suggested power to detect very small differences in rates of change among patients in intervention clinics relative to those in control clinics.^22^ Between CDS implementation and September 19, 2017, 8937 patients had an index visit and a mean (SD) of 5.8 (5.5) postindex visits (median, 5 postindex visits) in 1 of 76 randomized clinics retained for analysis (median, 88 patients per clinic; range, 20-423 patients per clinic).

## Results

A total of 45 clinics were randomized to the intervention group and 35 to the control group; 4 clinics were excluded for having fewer than 20 eligible patients, leaving 42 intervention clinics and 34 control clinics. In all, 8937 individuals with SMI (4922 women [55.1%]; mean [SD] age, 48.4 [13.5] years) made an index visit and at least 1 follow-up visit (Table 1; Figure). A total of 5901 participants (66.0%) had bipolar disorder, 1747 had schizoaffective disorder (19.5%), and 1289 had schizophrenia (14.4%). A total of 7483 individuals (83.7%) were White, 905 were Black (10.1%), 182 were Native American (2.0%), and 125 were Hispanic (1.4%). The mean (SD) 10-year total CV risk was estimated at 8.3% (8.8%), while the mean (SD) total modifiable CV risk was estimated at 3.7% (5.6%). Most patients’ 30-year CV risk estimates indicated 1 (4138 [46.3%]) or 2 or more (3602 [40.3%]) major CV risk factors that did not change for the better.

**Table 1.** Patient Characteristics and CV Risk at Index Visit by System and Treatment Group

Characteristic	All		Site A		Site B		Site C	
	Control	CDS	Control	CDS	Control	CDS	Control	CDS
No.	4387	4550	2120	1807	1486	1818	781	925
Women, No. (%)	2392 (54.5)	2530 (55.6)	1157 (54.6)	962 (53.2)	797 (53.6)	1040 (57.2)	438 (56.1)	528 (57.1)
Age, mean (SD), y	48.1 (13.4)	48.6 (13.3)	47.8 (13.6)	47.3 (13.2)	47.8 (13.1)	48.3 (13.3)	49.6 (13.6)	52.0 (13.0)
Age group, No. (%)								
18-39 y	1265 (28.8)	1270 (27.9)	632 (29.8)	553 (30.6)	434 (29.2)	527 (29.0)	199 (25.5)	190 (20.5)
40-49 y	961 (21.9)	962 (21.1)	472 (22.3)	400 (22.1)	325 (21.9)	397 (21.8)	164 (21.0)	165 (17.8)
50-59 y	1194 (27.3)	1263 (27.8)	550 (25.9)	515 (28.5)	418 (28.1)	483 (26.6)	226 (28.9)	265 (28.6)
60-64 y	462 (10.5)	493 (10.8)	220 (10.4)	163 (9.0)	161 (10.8)	194 (10.7)	81 (10.4)	136 (14.7)
65-75 y	505 (11.5)	562 (12.4)	246 (11.6)	176 (9.7)	148 (10.0)	217 (11.9)	111 (14.2)	169 (18.3)
Race and ethnicity, No. (%)								
Asian	55 (1.3)	76 (1.7)	35 (1.7)	52 (2.9)	8 (0.5)	10 (0.6)	12 (1.5)	14 (1.5)
Black	390 (8.9)	515 (11.3)	287 (13.5)	329 (18.2)	39 (2.6)	73 (4.0)	64 (8.2)	113 (12.2)
Hispanic	72 (1.6)	53 (1.2)	53 (2.5)	31 (1.7)	6 (0.4)	11 (0.6)	13 (1.7)	11 (1.2)
Native American	69 (1.6)	113 (2.5)	19 (0.9)	15 (0.8)	41 (2.6)	98 (5.4)	9 (1.2)	0
White	3740 (85.3)	3743 (82.3)	1691 (79.8)	1361 (75.3)	1386 (93.3)	1614 (88.8)	663 (84.9)	768 (83.0)
Other or unknown^a^	133 (3.0)	103 (2.3)	88 (4.2)	50 (2.8)	12 (2.8)	23 (1.3)	33 (4.2)	30 (3.2)
SMI subgroup, No. (%)								
Schizoaffective	864 (19.7)	883 (19.4)	413 (19.5)	382 (21.1)	296 (19.9)	295 (16.2)	155 (19.8)	206 (22.3)
Schizophrenia	657 (15.0)	632 (13.9)	234 (11.0)	262 (14.5)	297 (20.0)	245 (13.5)	126 (16.1)	125 (13.5)
Bipolar	2866 (65.3)	3035 (66.7)	1473 (69.5)	1163 (64.4)	893 (60.1)	1278 (70.3)	500 (64.0)	594 (64.2)
ACC/AHA 10-y CV risk								
No.	3586	3708	1744	1500	1168	1377	674	831
Mean (SD)	8.1 (8.7)	8.5 (8.9)	7.8 (8.6)	8.1 (8.7)	8.6 (8.6)	8.5 (8.8)	8.2 (8.9)	9.1 (9.6)
25th Percentile	2.3	2.4	2	2.3	2.6	2.5	2.4	2.4
Median	5.2	5.4	4.8	5.2	5.9	5.5	5.1	5.8
75th Percentile	11.1	11.5	10.2	10.7	11.7	11.4	11.4	12.7
Aged >39 y with no CHD								
No.	2569	2704	1221	1059	845	994	503	651
Mean (SD)	9.1 (8.7)	9.5 (9.1)	8.8 (8.7)	9.3 (9.1)	9.5 (8.7)	9.3 (8.7)	8.9 (8.7)	10.1 (9.9)
25th Percentile	3.0	3.2	2.6	3.1	3.4	3.2	2.8	3.3
Median	6.3	6.5	6.1	6.5	7.1	6.4	5.9	6.8
75th Percentile	12.5	12.7	12.1	12.4	13	12.3	12.6	13.5
Framingham 10-y CV risk								
No.	4387	4550	2120	1807	1486	1818	781	925
Mean (SD)	7.1 (8.1)	7.3 (8.2)	6.7 (7.8)	6.9 (8.0)	7.7 (8.5)	7.4 (8.2)	7.3 (8.2)	8 (8.7)
25th Percentile	1.8	1.9	1.7	1.6	2.1	2.0	1.9	2.0
Median	4.0	4.1	3.7	3.8	4.5	4.1	4.1	4.7
75th Percentile	9.1	9.9	8.3	9.0	10.1	9.9	9.7	11.1
Aged >39 y with no CHD								
No.	2925	3077	1395	1184	984	1194	546	699
Mean (SD)	8.6 (8.6)	8.8 (8.6)	8.1 (8.3)	8.5 (8.6)	9.3 (9.0)	8.8 (8.4)	8.4 (8.4)	9.2 (8.9)
25th Percentile	2.6	2.7	2.4	2.5	3.0	2.8	2.5	2.8
Median	5.6	5.7	5.1	5.6	6.4	5.7	5.4	6.1
75th Percentile	11.6	12.1	10.9	11.3	12.6	12.4	11.3	12.9
Modifiable CV risk								
Mean (SD)	3.6 (5.5)	3.7 (5.7)	3.4 (5.6)	3.6 (5.5)	4.1 (5.6)	3.9 (5.8)	3.3 (5)	3.4 (5.5)
25th Percentile	0.3	0.3	0.3	0.3	0.5	0.5	0.3	0.3
Median	1.6	1.6	1.4	1.5	2.1	2.0	1.4	1.3
75th Percentile	4.3	4.3	3.9	4.3	5.1	4.4	3.9	4.1
30-y (Lifetime) CV risk								
Aged <60 y, No.	2685	2733	1310	1181	884	1011	491	541
Risk factors, %								
All optimal	2.5	2.5	2.6	2.7	2.1	2.4	2.9	2.4
≥1 Not optimal	7.5	7.4	9.2	7.5	4.9	6.8	7.7	8.5
≥1 Elevated	3.9	3.8	4.5	3.6	3.5	3.5	3.3	5.2
1 Major	46.3	46.3	48.9	46.7	41.9	44.3	47.3	45.3
≥2 Major	39.8	40.7	34.8	39.5	47.6	43.0	38.9	38.6
Aged <60 y with no CHD, No.	2585	2627	1262	1140	847	963	476	524
Risk factors, %								
All optimal	2.6	2.6	2.7	2.8	2.2	2.5	2.9	2.5
≥1 Not optimal	7.7	7.6	9.4	7.7	5	7	8	8.6
≥1 Elevated	4.1	4.0	4.7	3.7	3.7	3.6	3.4	5.3
1 Major	46.9	46.2	49.5	47.6	42.5	44.9	47.9	45.6
≥2 Major	38.7	39.6	33.7	38.2	46.6	42.1	37.8	38
Current smoking, No. (%)	2027 (46.2)	2131 (46.8)	930 (43.9)	840 (46.5)	782 (52.6)	982 (54.0)	315 (40.3)	309 (33.4)
Body mass index^b^								
Mean (SD)	32.8 (8.0)	32.6 (7.8)	32.6 (7.9)	32.2 (7.6)	32.8 (7.9)	32.7 (8.1)	32.9 (8.3)	32.8 (7.6)
25th Percentile	27.3	27.2	27.4	27	27.2	27.2	27.4	27.3
Median	31.6	31.5	31.4	31.4	31.9	31.6	31.4	31.5
75th Percentile	36.9	36.8	36.6	36.4	37.1	36.9	37.3	37.2
Systolic blood pressure, mm Hg								
Mean (SD)	124.2 (16.5)	124.4 (16.6)	123.9 (16.7)	124.6 (17.2)	125.1 (16.6)	124.4 (16.1)	123.2 (15.9)	124.3 (16.5)
25th Percentile	112	113	112	113	114	114	111	112
Median	123	124	123	124	123	124	122	123
75th Percentile	134	134	133	135	135	134	132	134
Diastolic blood pressure, mm Hg								
Mean (SD)	78.3 (11.4)	78.3 (11.4)	79.0 (11.7)	79.9 (11.9)	77.9 (10.8)	77.7 (10.5)	77.0 (11.5)	76.5 (11.8)
25th Percentile	70	70	71	72	70	71	70	69
Median	78	78	79	80	78	78	76	76
75th Percentile	85	86	86	87	84	84	84	84
Blood pressure <140/90 mm Hg, No. (%)	3712 (84.6)	3816 (83.9)	1744 (82.3)	1417 (78.4)	1296 (87.2)	1590 (87.5)	672 (86.0)	809 (87.5)
LDL-C, mg/dL								
Mean (SD)	104.6 (35.6)	104.9 (35.4)	104.3 (34.8)	102.1 (34.4)	105.3 (36.8)	106.9 (36.3)	104.1 (35.7)	106.7 (35.4)
25th Percentile	80	80	80	78	79	81	79	81
Median	102	103	102	100	102	104	101	104
75th Percentile	126	126	126	122	128	130	126	127
HbA_1c_ among those with diabetes								
No.	866	947	375	376	309	337	182	234
Mean (SD)	7.3 (1.8)	7.1 (1.7)	7.4 (1.9)	7.2 (1.6)	7.2 (1.7)	7.1 (1.6)	7.4 (1.9)	7.1 (1.7)
25th Percentile	6.1	6.0	6.1	6.1	6.1	6.0	6.2	6.0
Median	6.9	6.7	6.9	6.8	6.8	6.7	6.9	6.6
75th Percentile	7.9	7.7	7.9	7.9	7.8	7.6	8.3	7.6
HbA_1c_, No. (%)								
<7%	464 (53.6)	546 (57.7)	191 (50.9)	206 (54.8)	177 (57.3)	201 (59.6)	96 (52.7)	139 (59.4)
7% to <8%	186 (21.5)	205 (21.6)	91 (24.3)	81 (21.5)	59 (19.1)	71 (21.1)	36 (19.8)	53 (22.6)
8% to <9%	83 (9.6)	63 (6.7)	37 (9.9)	28 (7.4)	28 (9.1)	20 (5.9)	18 (9.9)	15 (6.4)
≥9%	133 (15.4)	133 (14.0)	56 (14.9)	61 (16.2)	45 (14.6)	45 (13.4)	32 (17.6)	27 (11.5)

Abbreviations: ACC/AHA, American College of Cardiology/American Heart Association; CDS, clinical decision support; CHD, coronary heart disease; CV, cardiovascular; HbA_1c_, hemoglobin A_1c_; LDL-C, low-density lipoprotein cholesterol; SMI, serious mental illness.

SI conversion factors: To convert LDL-C to millimoles per liter, multiply by 0.0259; and HbA_1c_ to proportion of total hemoglobin, multiply by 0.01.

^a^ Included patients who were Pacific Islanders and patients for whom no race or ethnicity data were recorded in the electronic health record.

^b^ Calculated as weight in kilograms divided by height in meters squared.

**Figure.**
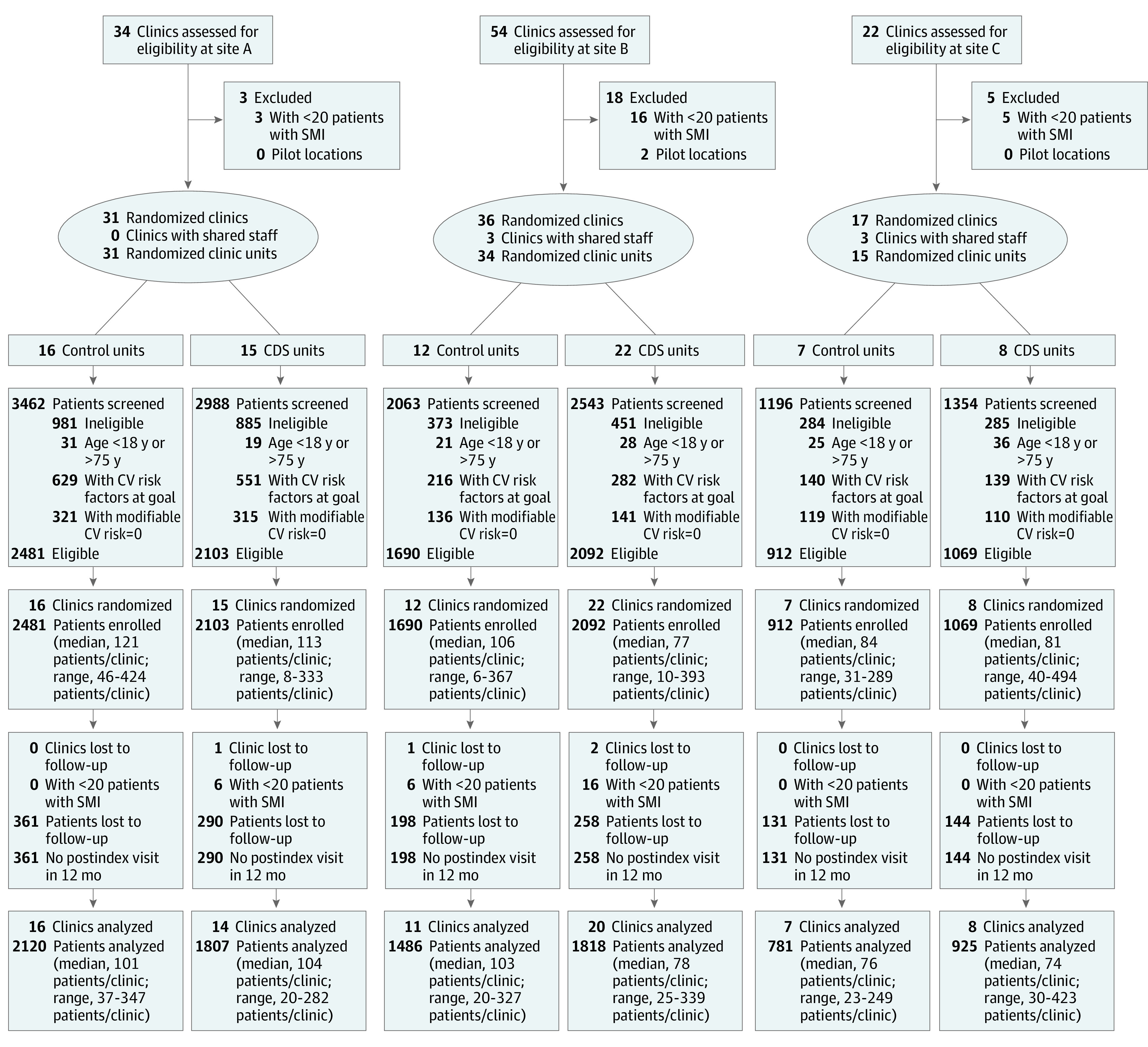
Study Consolidated Standards of Reporting Trials Diagram CDS indicates clinical decision support; CV, cardiovascular; and SMI, serious mental illness.

Intervention patients had an estimated 1% decrease in total modifiable CV risk (rate ratio [RR], 0.99; 95% CI, 0.98-1.01) over 12 months, while control patients had an estimated 4% increase (RR, 1.04; 95% CI, 1.02-1.05), for a net 4% lower increase in total modifiable CV risk among patients in intervention clinics relative to those in control clinics (relative RR, 0.96; 95% CI, 0.94-0.98) (Table 2).

**Table 2.** Total American College of Cardiology/American Heart Association 10-Year CV Risk at Index Visit, Model-Estimated Modifiable CV Risk at Index Visit and 12 Months, and Rate of Change by Time and Treatment × Time for Patients Overall and by Subgroup^a^

Characteristic	No.	Total CV risk at index visit, mean	Total modifiable CV risk			
			LS mean		Rate of change	
			Index visit	12 mo	RR for time	RR (95% CI) for treatment × time
All						
Control	4387	8.12	1.86	1.93	1.04	0.96 (0.94-0.98)
CDS	4550	8.49	1.90	1.89	0.99	
**SMI subtype**						
Bipolar disorder						
Control	2866	7.39	1.64	1.73	1.05	0.96 (0.94-0.99)
CDS	3035	7.96	1.69	1.72	1.02	
Schizoaffective disorder						
Control	855	8.68	2.23	2.29	1.03	0.94 (0.90-0.98)
CDS	877	8.73	2.20	2.13	0.97	
Schizophrenia						
Control	657	10.33	2.84	3.18	1.12	0.92 (0.85-0.99)
CDS	632	10.32	3.14	3.24	1.03	
**Total modifiable CV risk at index visit**						
0%-2%						
Control	2389	4.25	0.65	0.88	1.36	0.96 (0.92-0.99)
CDS	2467	4.37	0.66	0.86	1.30	
2%-5%						
Control	1048	7.77	2.99	3.26	1.09	0.96 (0.93-0.99)
CDS	1084	8.10	3.04	3.19	1.05	
5%-10%						
Control	495	12.56	6.06	5.61	0.93	0.97 (0.93-1.01)
CDS	544	12.90	6.14	5.52	0.90	
>10%						
Control	455	20.27	13.02	10.67	0.82	0.96 (0.92-0.99)
CDS	455	21.91	13.58	10.69	0.79	
**Sex**						
Female						
Control	2392	6.10	1.69	1.84	1.09	0.95 (0.92-0.97)
CDS	2530	6.28	1.73	1.78	1.03	
Male						
Control	1994	10.54	2.12	2.10	0.99	0.96 (0.94-0.99)
CDS	2020	11.24	2.18	2.08	0.95	
**Age group, y**						
18-29						
Control	466	3.02	1.57	1.72	1.09	0.89 (0.81-0.98)
CDS	439	3.07	1.48	1.44	0.97	
30-39						
Control	799	3.71	1.60	1.73	1.08	0.97 (0.92-1.03)
CDS	831	3.67	1.65	1.74	1.05	
40-49						
Control	961	5.01	1.65	1.72	1.04	0.98 (0.94-1.03)
CDS	962	4.94	1.74	1.78	1.02	
50-59						
Control	1194	7.91	1.90	1.98	1.05	0.93 (0.90-0.96)
CDS	1263	8.47	2.04	1.99	0.97	
60-75						
Control	967	15.37	2.34	2.35	1.00	0.97 (0.94-1.01)
CDS	1055	15.58	2.26	2.20	0.98	
**Race and ethnicity**						
Asian						
Control	55	7.59	1.95	1.69	0.86	1.11 (0.93-1.33)
CDS	76	7.10	1.79	1.71	0.96	
Black						
Control	390	9.43	2.15	2.33	1.09	0.93 (0.88-0.98)
CDS	515	10.55	2.17	2.19	1.01	
Hispanic						
Control	72	6.87	1.94	2.00	1.03	0.87 (0.75-1.01)
CDS	53	9.06	2.36	2.12	0.90	
Native American						
Control	68	7.07	2.13	1.97	0.93	0.98 (0.85-1.13)
CDS	110	7.82	2.39	2.17	0.91	
White						
Control	3736	8.03	1.83	1.90	1.04	0.96 (0.94-0.98)
CDS	3740	8.27	1.86	1.85	1.00	
Other and unknown^b^						
Control	66	8.73	1.76	1.62	0.92	1.16 (0.94-1.43)
CDS	56	6.31	2.01	2.14	1.07	

Abbreviations: CDS, clinical decision support; CV, cardiovascular; LS, least-squares; RR, rate ratio; SMI, serious mental illness.

^a^ Analyses adjusted for age, sex, and index visit outcome value.

^b^ Includes Pacific Islanders and all patients for whom race or ethnicity was not indicated in the electronic health record.

Despite the overall positive treatment effect, there were no significant treatment effects for individual modifiable CV risk factors, except for BMI, for which small differences favored the control group (Table 3). Small nonsignificant changes in low-density lipoprotein cholesterol level, HbA_1c_ level, and smoking favored the intervention group.

**Table 3.** Model-Estimated Rate of Change From Index Visit to 12 Months After Index Visit in Individual Modifiable CV Risk Factors and Obesogenic Medication Use Among Patients in CDS Clinics Relative to Control Clinics

Characteristic	Risk ratio or difference in difference (95% CI)^a^^,^^b^			
	All	Site A	Site B	Site C
Quit smoking^a^	1.11 (0.92 to 1.33)	1.19 (0.91 to 1.55)	1.04 (0.77 to 1.41)	1.09 (0.48 to 2.44)
Body mass index^b^	0.07 (0.01 to 0.13)	0.09 (0.01 to 0.17)	0.04 (−0.08 to 0.15)	0.19 (−0.06 to 0.44)
Low-density lipoprotein cholesterol^b^	−0.92 (−4.65 to 2.80)	−2.96 (−7.90 to 1.99)	4.47 (−2.70 to 11.63)	−4.86 (−22.94 to 13.22)
Systolic blood pressure^b^	1.03 (−1.04 to 3.09)	−0.26 (−3.29 to 2.77)	3.64 (−0.30 to 7.58)	−1.65 (−8.25 to 4.96)
Hemoglobin A_1c_^b^	−0.04 (−0.13 to 0.05)	−0.03 (−0.15 to 0.09)	−0.06 (−0.23 to 0.11)	−0.11 (−0.46 to 0.24)
Obesogenic SMI medication discontinuation^a^	1.02 (0.81 to 1.29)	1.03 (0.76 to 1.39)	0.92 (0.58 to 1.47)	1.15 (0.56 to 2.38)

Abbreviations: CDS, clinical decision support; CV, cardiovascular; SMI, serious mental illness.

^a^ Risk ratio (95% CI), adjusted for sex, age at index visit, log (year of index visit to last visit), site, and balancing factors.

^b^ Difference in difference (95% CI), adjusted for sex, age at index visit, index visit value, site, and balancing factors.

Change in modifiable CV risk varied by patient subgroup (Table 2). The effectiveness of the intervention varied by SMI subtype, with rate of change in intervention vs control patients 4% slower (RR, 0.96; 95% CI, 0.94-0.99) for patients with bipolar disorder, 6% slower (RR, 0.94; 95% CI, 0.90-0.98) for patients with schizoaffective disorder, and 8% slower (RR, 0.92; 95% CI, 0.85-0.99) for patients with schizophrenia. The intervention was similarly effective for women (RR, 0.95; 95% CI, 0.92-0.97) and men (RR, 0.96; 95% CI, 0.94-0.99). Change in total modifiable CV risk favored intervention patients aged 18 to 29 years (RR, 0.89; 95% CI, 0.81-0.98) and 50 to 59 years (RR, 0.93; 95% CI, 0.90-0.96) but not other ages. The intervention benefited patients who self-identified as Black (RR, 0.93; 95% CI, 0.88-0.98) or White (RR, 0.96; 95% CI, 0.94-0.98) patients but not Asian (RR, 1.11; 95% CI, 0.93-1.33), Native American (RR, 0.98; 95% CI, 0.85-1.13), or Hispanic (RR, 0.87; 95% CI, 0.75-1.01) patients or those of other or unknown race (RR, 1.16; 95% CI, 0.94-1.43). Patients of racial and ethnic minority groups generally had higher model-estimated total modifiable CV risk than White patients at the index visit and 12 months after the index visit. Patient and clinician handouts were printed at 61.1% of eligible encounters (37 079 of 60 685) in intervention clinics, and the rate of change in CV risk was not associated with print rates (eTables 3 and 4 in Supplement 2).

There were no clinician reports of adverse events. Surveillance of safety data aggregated from EHRs found no significant treatment × time effects for any monitored safety outcome (eTables 5-7 in Supplement 2).

## Discussion

This cluster randomized primary care CDS intervention produced mostly nonsignificant improvements in individual modifiable CV risk factors that, when summed together, resulted in 4% lower total modifiable CV risk among adults with SMI at 12 months. The CDS intervention favored patients who were 18 to 29 years of age or 50 to 59 years of age, Black, or White.

To our knowledge, this is the first randomized clinical trial shown to improve CV health in a large population of US outpatients with SMI. Few previous studies have addressed CV risk among those with SMI, and those that did had smaller sample sizes (a few hundred at most) or focused on a single CV risk factor.^30^ Furthermore, effects on overall CV risk have been rarely reported.^30^ One US study randomized 269 outpatients with SMI (including depression) and 1 CV risk factor not at the goal set by the ACC/AHA guidelines to counseling and care coordination.^31^ Intervention patients achieved a 12.7% relative reduction in the 10-year Framingham Risk Score at 19 months compared with control patients. In a US study of collaborative care for 118 veterans with bipolar disorder, intervention patients had decreased systolic and diastolic blood pressure but not cholesterol level at 24 months.^32^ A British cluster randomized primary care trial involving personalized goal setting for 327 patients with SMI showed no difference in the primary outcome of total cholesterol level or a secondary outcome of total CV risk at 12 months.^33^ Finally, a Danish randomized trial of lifestyle coaching plus care coordination for 428 patients with schizophrenia and central obesity had no effect on 10-year CV risk, BMI, systolic blood pressure, HbA_1c_ level, or smoking status at 12 months.^34^

Our finding of a 4% relative decrease in modifiable CV risk in intervention compared with control patients was modest yet clinically significant, particularly given the relatively low intensity of the intervention and the intention-to-treat analysis. For this population of patients with a baseline 10-year CV risk of 8%, approximately 80 of 1000 patients would be predicted to have a myocardial infarction or stroke in the next 10 years. The relative 4% decrease that we observed with the intervention would result in an estimated 7.7% 10-year CV risk, or approximately 77 of 1000 events, potentially preventing 3 events per 1000 patients. Thus, although a 4% lower CV risk appears modest, on a population level it is clinically meaningful, especially in the context of an observed upward trend in 10-year risk in the control group and a stable trend in the intervention group.

Similar to studies of total CV risk, studies focused on single CV risk factors among patients with SMI have had mixed results. In a UK study of 526 outpatients, smoking cessation rates were higher for intervention patients than for control patients at 6 months (14% vs 6%; risk difference, 7.7%; 95% CI, 2.1%-13.3%), but this effect was nonsignificant at 12 months, the primary end point.^35^ In a US trial of a behavioral weight-loss intervention for 291 outpatients with SMI (including depression), there was a mean between-group difference in weight of 3.2 kg (*P* = .002) favoring the intervention at 18 months.^36^ Although the intensity of our intervention was lower than in these studies, our CDS intervention reached thousands of patients with SMI and addressed multiple modifiable CV risk factors, resulting in lower total modifiable CV risk.

There were significant differences in intervention effectiveness by race and ethnicity. Black and White intervention patients had lower total modifiable CV risk relative to control patients at 12 months, while patients in other racial or ethnic groups did not. Patients in racial and ethnic minority groups started and ended the intervention with higher estimated modifiable CV risk than did White patients, consistent with the cumulative effects of structural racism on cardiometabolic risk.^37,38^ Existing research on CV risk of patients in racial and ethnic minority groups with SMI have been scarce. A 2014 literature review was limited by a small number of studies with small sample sizes and varied designs and methods.^39^ Despite these limitations, the authors found that Black patients with SMI, especially Black women with SMI, were at greater risk for obesity and, along with Hispanic patients, for diabetes, especially when exposed to antipsychotic medications. We encourage future studies to report outcomes by race and ethnicity and further address CV health disparities for patients in racial and ethnic minority groups with SMI.

Clinicians were asked to address CV risk starting at 18 years of age rather than waiting until the more standard age of 40 years. This decision was supported by findings that patients with SMI have higher CV burden at younger ages, resulting in shortened life spans.^2^ The intervention had significant effects for patients who were 18 to 29 years of age, resulting in a relative 11% decrease in change in total modifiable CV risk at 12 months. This finding is important as evidence emerges of improved lifelong outcomes when CV risk for patients with SMI is addressed earlier in the life span.^40^ Our findings support the value in addressing elevated 30-year risk with evidence-based interventions.

Some may question the usability and sustainability of a low-intensity CDS intervention. Clinicians have previously reported ignoring CDS owing to inconsequential alerts, distrust, alert fatigue, or workflow disruption.^41,42,43^ Our CDS intervention was designed to fit with rooming staff workflows to avoid interference with clinician workflows, leading to print rates of the CDS output at 61.1% of targeted visits. Furthermore, at the request of health care system leaders, the CDS was turned on for all primary care clinics at 2 participating health care systems the day after the study ended. The CDS has since been printed at 73.7% of eligible encounters, supported by monthly feedback of print rates to clinic leadership. These findings highlight the usability and sustainability of CDS interventions.

### Limitations

This study has some limitations. It was conducted in 3 Midwestern integrated health care systems, and the results may not be generalizable to other settings. This pragmatic intervention was implemented on top of usual care, with patients returning to the clinic at intervals deemed appropriate by their care team, and 12-month outcomes were derived from available data. We relied on EHR diagnoses to identify patients with SMI, and some patients may have been misclassified.

## Conclusions

In this large cluster randomized clinical trial of an EHR-integrated CDS tool, intervention patients had 4% lower change in total modifiable CV risk at 12 months compared with control patients. Intervention patients with SMI who were 18 to 29 years of age or 50 to 59 years of age, Black, or White had significantly lower change in modifiable CV risk at 12 months compared with control patients. The effects of our intervention on young adults with SMI highlights the value of using 30-year CV risk estimates to prompt primary care intervention for this population. Our intervention helped slow the otherwise increasing CV health disparities for Black patients with SMI, and we encourage future interventions to focus on further improving CV health in disadvantaged populations.

## Notes

Supplement 1.
Trial Protocol

Supplement 2. **eTable 1.** Serious Mental Illness (SMI) Diagnostic Codes **eTable 2.** Potentially Obesogenic Medications for Serious Mental Illness (SMI) **eTable 3.** Intervention Dose, ie, the Number and Percent of Visits (Index and Post-Index) at Which the CDS Was Printed by System and Treatment Group **eTable 4.** Rate Ratios Estimating Rates of Change in Total Modifiable CV Risk From Index to 12 Months Post-Index Among Patients in Intervention Clinics by Print Rate Subgroups **eTable 5.** Emergency Department Visit Rates by Health System and Treatment Group in the Year Prior to the First Visit (Pre) and Time Between First Visit and 9/19/2018 (Post) **eTable 6.** Inpatient Stay Rates by Health System and Treatment Group in the Year Prior to the First Visit (Pre) and Time Between First Visit and 9/19/2018 (Post) **eTable 7.** Suicide Attempt Rates by Health System and Treatment Group in the Year Prior to the First Visit (Pre) and Time Between First Visit and 9/19/2018 (Post)

Supplement 3.
Data Sharing Statement
