# Supplementary material for: Effect of Clinical Decision Support on Cardiovascular Risk Among Adults With Bipolar Disorder, Schizoaffective Disorder, or Schizophrenia: A Cluster Randomized Clinical Trial
Source: JAMA Netw Open. 2022 Mar 7;5(3):e220202. doi: 10.1001/jamanetworkopen.2022.0202 (PMC8902652; doi:10.1001/jamanetworkopen.2022.0202)
Supplement: Supplement 1. — Trial Protocol [file jamanetwopen-e220202-s001.pdf]

Reducing Cardiovascular Risk in Adults with Serious Mental Illness

NCT # 02451670

10.23.2014

Project Narrative

People with serious mental illness (SMI; schizophrenia, schizoaffective disorder, or bipolar disorder) die an average of 25 years earlier than their peers, and cardiovascular (CV) disease is the leading cause of death. Primary care providers are not adequately aware of the significantly increased CV risk in patients with SMI, and, even when CV risk factors are identified, often do not take appropriate clinical actions. This project will test the ability of an electronic medical record-based clinical decision support system to help primary care providers identify, provide more appropriate care for, and control CV risk factors for patients with SMI.

Project Summary

People with serious mental illness (SMI) (schizophrenia, schizoaffective disorder, bipolar disorder) die 25 years earlier than their peers, and cardiovascular (CV) disease is the leading cause of death. Primary care providers (PCPs) often are not aware of the significantly increased CV risk in patients with SMI and, even when CV risk factors are identified, appropriate clinical actions often are not provided. Electronic medical record (EMR)-based clinical decision support (CDS) delivered at a clinical encounter may be a powerful tool to help primary care providers (PCPs) identify and control CV risk factors in patients with SMI.

This proposal will adapt a point-of-care EMR-based CDS system (CV Wizard) to help PCPs identify, provide appropriate care for, and control CV risk factors for patients with SMI. CV Wizard is designed to educate PCPs about the increased risk of CV disease and mortality in people with SMI, identify elevated CV risk factors in patients with SMI, prioritize these CV risks based on how much improvement in CV risk a patient would experience if the CV risk factor was adequately addressed, recommend specific medications and other interventions to decrease each elevated CV risk factor, and provide this information in an easy-to-understand format for both patients with SMI and their PCPs. For those patients who are on the SMI medications most associated with weight gain, they will be able to work with a psychiatric care manager who, under the supervision of the patient's treating psychiatrist and with the consent of the patient, will switch the SMI medication to one less associated with weight gain. The effectiveness of this intervention will be assessed in a clinic-randomized trial with 52 primary care clinics, 150 PCPs, and about 2,250 adults with SMI. We hypothesize that, relative to patients with SMI receiving care in control clinics, those in the CV Wizard clinics will have (a) reduced total modifiable CV risk; (b) better control of six individual modifiable CV risk factors, including blood pressure, lipids, tobacco use, aspirin use, overweight/obesity, and, for those with diabetes, glucose control, and (c) lower rates of prescriptions of SMI medications that are most associated with weight gain. In secondary analyses, we will also explore the impact of CV Wizard and care management on CV risk factor identification, treatment initiation and intensification; medication adherence; outpatient and inpatient utilization; risky prescribing events; and CV events.

This study targets an important area of research that is a priority for the National Institute of Mental Health, our health system partners, and our external stakeholder advisory board; leverages previous infrastructure investments in the Mental Health Research Network; and capitalizes on the expertise of our researchers. Developing an effective EMR-driven point-of-care CDS strategy that identifies and prioritizes available treatment options to better address uncontrolled CV risk factors in adults with SMI is a critical next step to improving the health and reducing the CV risk of this medically underserved population.

A Randomized Clinical Trial to Reduce Cardiovascular Risk in Adults with SMI

Specific Aims

People with serious mental illness (SMI) (schizophrenia, schizoaffective disorder, bipolar disorder) die, on average, 25 years earlier than their peers. Cardiovascular (CV) disease is the predominant cause.[1] Primary care providers (PCPs) are not adequately aware of the significantly increased CV risk in patients with SMI and, even when they do identify elevated CV risk factors, often do not take appropriate clinical actions.[2-4] Electronic medical record (EMR)-based clinical decision support (CDS) can identify at-risk patients with SMI and systematically prompt more effective treatment of their CV risk factors, but its potential has been largely untapped. The objectives of this project are to improve CV risk factor care in patients with SMI through a pragmatic trial of a point-of-care EMR-based CDS (referred to as “CV Wizard”) and a nurse care manager. The trial will be conducted in over 50 “real world” primary care clinics in three large healthcare systems.

In Phase 1 of the project, we will: (a) extend our EMR CDS algorithms, which were successfully used in a population of adults with diabetes,[5] including a small subset of those with modifiable CV risk and SMI, to (i) identify people with SMI and (ii) identify elevated CV risk and available treatment options; (b) develop algorithms prioritizing these options for a specific patient at each clinic visit (including possible referral to a psychiatric nurse care manager to assist with changing the antipsychotic regimen); (c) develop an effective interface to communicate this information to patients and PCPs, including specific “what drug, what dose” recommendations; (d) train nurse care managers to act liaisons between primary care and behavioral health, and assist patients in switching their obesogenic SMI medications to those with less risk, when appropriate; (e) tailor the CV Wizard to interface correctly with the EMR at each study site; and (f) develop office systems that orchestrate nurse and PCP actions to reinforce consistent ongoing use of this novel intervention.

In Phase 2, primary care clinics will be randomly assigned to receive or not receive the intervention with staggered fashion, allowing each site to learn lessons from the site(s) implementing before it and increasing efficiency. This randomized trial will test the hypothesis that compared with usual care, the intervention will significantly reduce CV risk and improve care and control of specific CV risk components (blood pressure, lipid levels, glycosylated hemoglobin, smoking, obesity, aspirin use) in adults with SMI.

Phase 3 of the project includes (a) analysis of clinical effectiveness, (b) secondary analyses to identify mediators and modifiers of the impact of the intervention, and (c) dissemination and spread of the most successful aspects of the intervention model. To achieve these objectives, we propose these specific aims:

Primary Aim. To assess the impact of CV Wizard and psychiatric nurse care management on CV risk factor control and appropriate SMI medication use in adults with SMI.

> **Hypothesis 1.** Adults with SMI receiving care in intervention clinics will have lower total modifiable CV risk 12 months post-index than those receiving care in control clinics.
>
> **Hypothesis 2.** Adults with SMI receiving care in intervention clinics will have better control of specific modifiable CV risk factors 12 months post-index than those receiving care in control clinics.
>
> **Hypothesis 3**. Adults with SMI receiving care in intervention clinics will have lower rates of prescriptions for obesogenic SMI medications 12 months post-index than those receiving care in control clinics.

Secondary Aim. To explore the impact of CV Wizard and psychiatric nurse care management on CV risk factor identification, treatment initiation and intensification; medication adherence; outpatient and inpatient utilization; risky prescribing events; and CV events.

This project targets an important clinical domain that is a priority of NIMH, our health system partners, and our external stakeholder advisory board, leverages previous MHRN infrastructure investments, and capitalizes on the expertise of MHRN researchers. It also builds on a decade of our team’s NIH-funded work in CV disease, diabetes, mental illness, and CDS development and implementation. Developing an effective EMR-driven point-of-care CDS strategy that identifies and prioritizes available treatment options to better address uncontrolled CV risk factors in adults with SMI is a critical next step to improving the health and reducing the CV risk of this medically underserved population. In addition to data from the EMR and claims, we will interview adults with SMI about other potential contributors to CV risk and their experiences with the intervention. Therefore, regardless of the specific results of the project, we will collect crucial information to improve the CV health and inform future interventions in this area of immense importance to the health of adults with SMI.

Research Strategy

SIGNIFICANCE

People with serious mental illness (SMI) die, on average, 25 years younger than age- and gender-matched patients without SMI.[1] Medical illness accounts for 60% of premature death in SMI, while suicide and injury account for 30% to 40%.[1] Cardiovascular (CV) disease is the leading cause of death in adults with SMI.[1] People with schizophrenia die of CV disease at 2.3 times the rate in the general population, and more than two-thirds of people with schizophrenia die of coronary heart disease.[6, 7] The relative risk for individual CV risk factors in patients with SMI compared with the general population is 1.5-2 for obesity, 2-3 for smoking, 2 for diabetes, and up to 5 for dyslipidemia.[8, 9] Druss et al found that the presence of a mental disorder was associated with a 19% increase in 1-year risk of mortality (HR 1.19; 95% CI,1.04-1.36), and that this excess mortality was accounted for in large part by deficits in quality of preventive medical care.[10] In a study of patients enrolled in Minnesota health care programs from 2003 to 2007, women with SMI died at a median age of 63 years, compared with 85 years for controls; men with SMI died at a median age of 53 years, compared with 74 years for controls.[11] Examining the 387 people in this cohort who died of CV disease, patients with SMI died at a median age of 56 years, compared with 83 years in controls—an average of 27 years of life lost.[11]

Complex combinations of behavioral, pathophysiologic, care-delivery system, provider, and medication-related factors are likely involved in the association between SMI and CV disease. *Behavioral factors* include significantly increased rates of obesity, metabolic syndrome, sedentary lifestyle, poor diet, smoking, treatment nonadherence, and decreased likelihood of seeking or obtaining preventive or medical care in those with SMI.[1, 4, 12-20] *Pathophysiological factors* include increased platelet reactivity, endothelial dysfunction, autonomic dysfunction, hypercortisolemia, abnormal immune system activation, and increased QT-interval variability in adults with SMI, and elevated systolic blood pressure (SBP) in patients with bipolar disorder who are manic, all of which may promote heart disease via pro-atherogenic, pro-ischemic, or pro-arrhythmic processes.[21-24] *Care delivery system and provider factors* include provider discomfort in treating patients with mental illness, poor coordination and continuity of care among providers, and a decreased tendency to recognize and adequately treat risk factors for CV disease in patients with SMI. Although the American Diabetes Association published a consensus guideline for monitoring patients on antipsychotic medications, it is seldom followed.[1, 25] Patients with mental illness continue to receive substandard care, including lower rates of CV procedures once medical care is sought.[26-28] *Medication-related factors* are complex, and the effects of SMI medication on CV disease are not well understood. Lithium and valproic acid, the most widely used mood stabilizers, are associated with weight gain. In addition, valproic acid has been associated with insulin resistance and hyperlipidemia,[22, 29] while lithium may affect cardiac conduction and renal function.[30] Second-generation antipsychotic medications are widely used, and many cause weight gain, insulin resistance, diabetes, dyslipidemia, and metabolic syndrome.[1] Antipsychotics have also been associated with QT-interval prolongation, orthostatic hypotension and, rarely, torsade de pointes.[31] Despite these cardiac effects, non-suicide and CV mortality rates appear to be lower in patients with SMI who take these medications long-term.[32, 33] This may be in part related to SMI medications' normalization of autonomic tone, which could decrease the risk of myocardial infarction.[34, 35]

In summary, those with SMI bear a huge excess risk of CV events and mortality that is multifactorial in origin. The problem deserves attention, and EMR-based CDS is a promising strategy to remedy several of the causes. Given the combination of behavioral and biomedical mechanisms linking SMI and CV risk, targets for interventions to reduce CV risk include both changing health behaviors (diet, physical activity, smoking) and improving medical control of modifiable risk factors (hyperglycemia, hyperlipidemia, hypertension, aspirin use). Recent research demonstrates that intensive behavioral interventions proven effective in the general population[36, 37] are also effective in people living with SMI.[38] The trial proposed here addresses a parallel question: Can a clinical decision support intervention proven effective in people with diabetes help to control cardiovascular risk factors in people living with SMI?

INNOVATION

Innovation in Adult SMI Care. Patients with SMI have exceedingly high CV event rates, which contribute to a shortened life expectancy. However, health care systems have only recently become aware of this and are now prioritizing doing something about it. However, they aren't sure what to do. The proposed EMR-based CDS (CV Wizard) represents a novel and innovative way to allow care systems to systematically address

uncontrolled CV risk factors in patients with SMI, who have multiple barriers to the receipt of standard recommended care for their medical illnesses. CV Wizard has potential to reduce disparities in care by increasing PCP awareness of this issue, providing timely identification and prioritized evidence-based treatment recommendations to control CV risk factors, and facilitate actively engaging patients in their care.

Innovations in Decreasing Use of Antipsychotics with Increased Cardiometabolic Risks. SMI medications contribute unequally to cardiometabolic risk. Certain SMI medications (most notably valproic acid, olanzapine, clozapine, thioridazine and chlorpromazine) can cause significant weight gain, often with associated changes in insulin resistance and lipid metabolism.[13, 39] Significant weight gain has also been reported, albeit to a smaller degree, with lithium, quetiapine and risperidone, while molindone, ziprasidone, aripiprazole, fluphenazine, haloperidol, pimozide, and loxapine appear to cause the least amount of weight gain in adults with SMI.[40-42] In the CAFÉ trial, patients who received olanzapine gained an average of 11.0 kg in 4 months, while those who received risperidone gained 6.4 kg, and those with quetiapine gained 5.5 kg.[43] Similarly, in the CATIE trial, 30% of patients randomized to olanzapine gained ≥7% of their body weight over 18 months or time of discontinuation, compared to 16% for quetiapine, 14% for risperidone, and 7% for ziprasidone.[35] Given these differences between SMI medications, our innovative intervention 1) uses existing EMR data to identify patients with SMI taking obesogenic SMI medications who have (a) increased their body weight ≥7% in the previous 12 months (similar to what was used in an HMORN pilot project,[44] allowing us to use these developed tools), or (b) who have a BMI≥35 kg/m^2; and 2) refers such patients to a psychiatric nurse care manager who will act as a liaison between the patient's primary care and behavioral health providers, implementing protocol-based medication transitions.

Innovative Model for Protocolized Pharmacotherapy. Traditional research models for delivering protocolized pharmacotherapy rely on specialized clinicians working in research clinics. The high cost and limited reach of this approach make it impractical for large-scale pragmatic trials, and such a model is certainly not viable for widespread dissemination or implementation. The proposed trial will implement and evaluate an alternative model for delivery of protocolized pharmacotherapy supported by a psychiatric nurse care manager and by workflow changes in the primary care clinic to support treatment recommendations.

Technical Innovations. The potential for EMR technology to improve clinical care and accelerate translation of evidence into practice has been widely recognized, but few studies of EMR-based CDS have shown positive results with respect to mental health care. Most previous EMR-based CDS interventions have failed for two principal reasons: (a) PCPs have not accessed the CDS information in a timely fashion, and (b) the CDS has been limited to simple prompts or reminders.[45, 46] This project addresses these limitations by applying these innovative approaches: 1) The CDS goes beyond simple computer prompts and reminders to provide more sophisticated, patient-specific CDS, considering previous CV risk factor values, diagnoses, and medications and making guideline-standardized yet personalized recommendations for a range of clinical actions at the point of care; 2) The CDS is provided to both the patient and primary care physician (PCP) early in the encounter and supported by changes in clinic workflows and team responsibilities previously shown to increase the likelihood that PCPs will use the CDS in a timely and sustained fashion;[47] 3) The CDS algorithms are implemented in a Web service linked to the EMR, simplifying clinical updates over time and increasing scalability of the intervention to other medical groups with other types of EMRs and additional mental health conditions in the future; 4) The CDS includes intuitive provider and patient interfaces developed to clearly identify high-benefit clinical or lifestyle actions that can substantively reduce CV risk and efficiently elicit patient treatment preferences. 5) The intervention is also novel in systematically recognizing patients with SMI and efficiently triggering the CDS tool.

Innovations Related to Future Research and Implementation. Finally, the prioritization and treatment algorithms and Web site developed in this project can support many other applications (e.g., mapping quality of care for people with SMI at the provider or clinic level, supporting "patient-direct" applications to inform patient selection of treatment options, or reaching patients through the Internet). This project builds on our team's previous successful efforts to use CDS to support goal-based guidelines[47] and extends that technology—already proven to improve blood pressure (BP) and glucose control in people with diabetes—to a new target population: people with SMI.

APPROACH

Preliminary Studies

Previous Experience: Our multidisciplinary research team has extensive experience and expertise in chronic disease, cardiology, primary care, mental health, CDS system design and implementation, and best-practices dissemination and implementation. The PI, Dr. Rossom, is an experienced psychiatrist who has led studies of the mortality associated with antipsychotics in elderly patients with dementia is an investigator on National Institutes of Health-sponsored studies of selective serotonin reuptake inhibitor use and suicide behaviors, a statewide initiative to implement best-practice models for depression care, a pragmatic trial to prevent suicide, the association between suicidal ideation and behavior, predictors of high-value treatments for mood disorders, and the association between chronic kidney disease and cognitive impairment (3U19CA079689, 1U19MH092201, 1UH2AT007755, 3U19M092201, 5R01MH080692, R01AG037551). She is also an investigator for a CMS-funded program involving care management of depression, diabetes, heart disease, and substance abuse across seven states (1C1CMS331048). Drs. O'Connor and Crain are conducting several federally funded randomized clinical trials of CDS tools for adults with major CV risk factors (HL102144, DK068314). Dr. Waring has research experience in primary care of mental illness, dementia, and rural healthcare, and is currently involved in a pilot study to develop a CDS system to improve primary care of dementia.[48-52] Dr. Owen-Smith has researched medical care for minority and other underserved populations and depression in medically ill patients.[53-56] Consultant Dr. Russell Luepker is a cardiologist with experience in evaluation and treatment of CV risk factors and disease and is chair of the Framingham Heart Study and Honolulu Heart Study Advisory Boards.[57-59] Consultant Dr. Ben Druss is a psychiatrist with experience in the evaluation and treatment of patients with SMI and a nationally recognized expert in the medical care of patients with mental illness.[10, 18, 60-66] Our team's previous and ongoing work in both mental health and cardiometabolic conditions (we have collectively written more than 500 articles and dozens of clinical guidelines) makes us uniquely well qualified to successfully conduct the proposed project.

Pilot Data: To assess the likelihood that the proposed intervention would be effective for adults with SMI, we examined the effect of an earlier CDS tool used with adults with diabetes at HealthPartners.[47] To provide pilot data for this proposal, we identified the subset of patients with diabetes and bipolar disorder (N=41) or schizophrenia (N=43). Due to the diabetes requirement, these patients had a mean age of 59 years, and 61% were female. In patients with bipolar disorder randomly assigned to the intervention group, A1c values declined by an absolute 0.8%, SBP by 9.4 mm Hg, diastolic BP (DBP) by 7.3 mm Hg, and low-density lipoprotein (LDL) cholesterol by 6.2 mg/dL at 18-months. Likewise, in patients with schizophrenia randomly assigned to the intervention group, A1c values declined by 0.7%, SBP by 11.8 mm Hg, DBP by 8.8 mm Hg, and LDL by 20.1 mg/dL. Patients with SMI in the control group started from different baseline values and had much smaller improvements in these CV risk factors (for control patients with bipolar disorder, A1c values increased by 0.2%, while SBP decreased by 3.3 mm Hg, DBP by 1.9 mm Hg, and LDL by 9.1 mg/dl at 18-month follow up; for control patients with schizophrenia, A1c values were unchanged, while SBP decreased by 3.9 mm Hg, DBP by 3.5 mm Hg, and LDL by 10.3 mg/dL over the same period). The notable improvement in CV risk measures in this small group of SMI patients with diabetes suggests that PCPs were able to use such CDS tools in the care of adults with SMI, that the patients with SMI were able to respond to this intervention in the desired way, and that this type of intervention may be strong enough to effect clinically significant improvements in the identification and control of key CV risk factors in adults with SMI.

Preliminary Assessment of Study Population: Preliminary data (**Table 1**) were collected at HealthPartners over 2 years to assess the feasibility of a significant reduction in CV risk among patients with SMI in the proposed trial. Although many SMI patients likely have BP<130/80 and LDL<130 mg/dL, about two-thirds have uncontrolled CV risk factors (especially smoking, hyperglycemia, and overweight/obesity) at any point.

Table 1. Description of Adults Age 18 and Older with SMI at HealthPartners Medical Group, 2009-2010.

Condition	N	Mean Age	Mean Weight (lbs)	Smoker (%)	Hyperglycemia (%)[2]	Dyslipidemia (mg/dL)[3]	Elevated BP (%)[4]
Bipolar disorder	1,871	43.3	196	39.5	15.4	2.9	7.7
Schizophrenia	469	50.4	193	45.6	25.4	3.2	10.5
Schizoaffective	570	48.2	206	40.3	27.4	3.7	8.4
Total	2,825	45.3	198	40.5	19.3	3.1	8.2

[1]Patients with missing data are not included in denominator. [2]Defined as A1c >= 6% or fasting glucose>=100 mg/dL on most recent observation. [3]Defined as LDL ≥130 mg/dL. [4]Defined as SBP ≥140 mmHg.

METHODS

Study Overview: In this cluster-randomized clinical trial, at least 52 primary care clinics at 3 Mental Health Research Network (MHRN) organizations will be blocked within organization on size and characteristics of patients with SMI and then randomly assigned 1:1 to CV Wizard or control clinics (**Figure 1**). All consenting PCPs will be allocated to the same study arm as their clinic, and the estimated 15 eligible adults with SMI under the care of each PCP will be allocated to the same study arm as their PCP.

Figure 1. Study Design for Group-Randomized Trial.

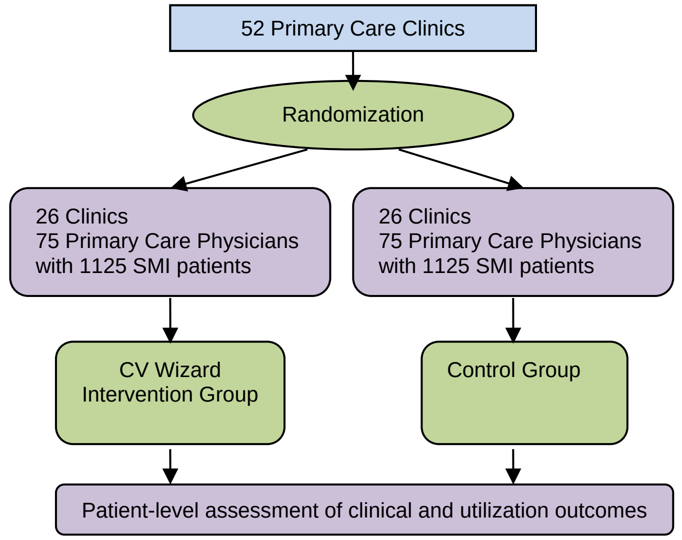

Study Sites: This study will be conducted at the primary care clinics of 3 organizations: **HealthPartners Medical Group (HPMG)** is a large multispecialty group that provides care to 300,000 adults at 24 clinics throughout the Twin Cities, including 2,825 adults diagnosed with SMI. Leaders at HPMG have committed 18 primary care clinics to the project.

Kaiser Permanente of Georgia (KPGA) provides care to 244,000 adults at 27 clinics, including about 3,400 with SMI. KPGA clinical leaders have committed 18 clinics with about 3100 patients with SMI to this project.

Essentia Health (EH) is a multispecialty integrated healthcare provider with clinics across Wisconsin, Minnesota, North Dakota, and Idaho. EH serves a primarily rural population of over 900,000, including 5,200 adults with SMI. Clinic leaders at Essentia have committed 16 clinics to this project. Like HPMG and KPGA, EH uses EpicCare EMR.

Study Participants: PCPs and Patients: To participate, **PCPs** must practice at a randomized primary care clinic and: (a) be a general internist, family physician, or adult-care non-obstetric nurse practitioner, (b) provide ongoing primary care for six or more adults with SMI in 2012, and (c) provide written informed consent to participate in the study. About 385 eligible PCPs practice at the clinics. We anticipate, based on experience, that we will successfully recruit 150 PCPs (75 per arm). This feasible recruitment target ensures that subgroup analyses (eg, based on gender, age) will be adequately powered and that the intervention strategy is suitable for use by a large, representative segment of PCPs.

To be included in the primary aim analysis, **patients** must meet the study criteria for diagnosed SMI (one inpatient or two outpatient ICD-9 codes for schizophrenia, schizoaffective disorder or bipolar disorder and: (a) be >17 years and <80 years, (b) have a Charlson comorbidity score of 3 or less, (c) be linked to a consented PCP, and (d) have at least one primary care visit with a consented PCP in the 12 months prior to clinic randomization. Based on preliminary HPMG data (**Table 1**), we estimate that 75% of adult patients with SMI visit a PCP at a participating clinic each year. We estimate that 95% of adults with SMI have Charlson scores ≤3, and 95% are ≤80. We anticipate up to 10% to 20% per year disenrollment and a 1% to 2% per year death rate of adults with SMI. Few patients with SMI at our study sites switch clinics, and our algorithms accurately match over 95% of patients to a regular PCP. Ultimately, after accounting for all exclusions, we anticipate about N≈2,100/140≈15 adults with SMI per eligible PCP will be study-eligible at HPMG. In the 52 randomized clinics, we anticipate including data from about N≈150 PCPs * 15 adults per PCP≈2,250 patients with SMI in the primary analysis. We will request a waiver of informed consent for patients from the IRB because the care recommendations in the CV Wizard intervention are limited to evidence-based care from current national and regional clinical guidelines. In the past, the IRB has waived consent in similar circumstances. Moreover, the CDS algorithms identify potentially risky treatment strategies (eg, use of a diuretic with lithium; full list to be developed in Phase 1), further reducing risks to patients with SMI. We describe in detail below how the proposed research satisfies criteria in 45 CFR 46 for waiver of informed consent.

Design, Implementation, and Use of CV Wizard Intervention. CV Wizard is rooted in a series of antecedent

studies that have developed more limited but successful forms of CDS.[5, 67] In our first phase, we will develop detailed algorithms that will operate in both the EMR and Web site to provide the necessary CDS (**Figure 2**).

Step 1: Identify Eligible SMI Patients and their CV Risk Factors and Prioritize Management of Any that are Uncontrolled: Web-based CDS algorithms estimate the patient's 10-year CV risk and quantify decreases in CV risk with appropriate management of out-of-control CV risk factors. CV risk is calculated based on the Framingham 10-year CV Disease Risk Equation.[68, 69] When measurements are not obtained within guideline-recommended timeframes,[25] CV Wizard prompts PCPs to obtain them. CV risk will be conceptualized as a vector with two components: (a) an irreversible CV risk vector related to age and gender (and their interaction), and (b) a modifiable CV risk vector related to uncontrolled CV risk factors (potentially reversible if these risk factors were controlled). Component (b), modifiable CV risk, is composed of a vector for each uncontrolled CV risk factor of interest: BP, lipids, smoking, BMI, aspirin use, and glucose. The magnitude of the vectors determines its priority, with priority 1 (**Figure 3**) assigned to the risk factor with the greatest potential reduction in CV risk, and so forth for all uncontrolled CV risk factors.

Figure 2. Schematic Representation of Prioritized Clinical Decision Support (CV Wizard) Intervention Showing How Open Treatment Options are Identified, Prioritized, and Presented to Patients and Primary Care Physicians (PCPs)

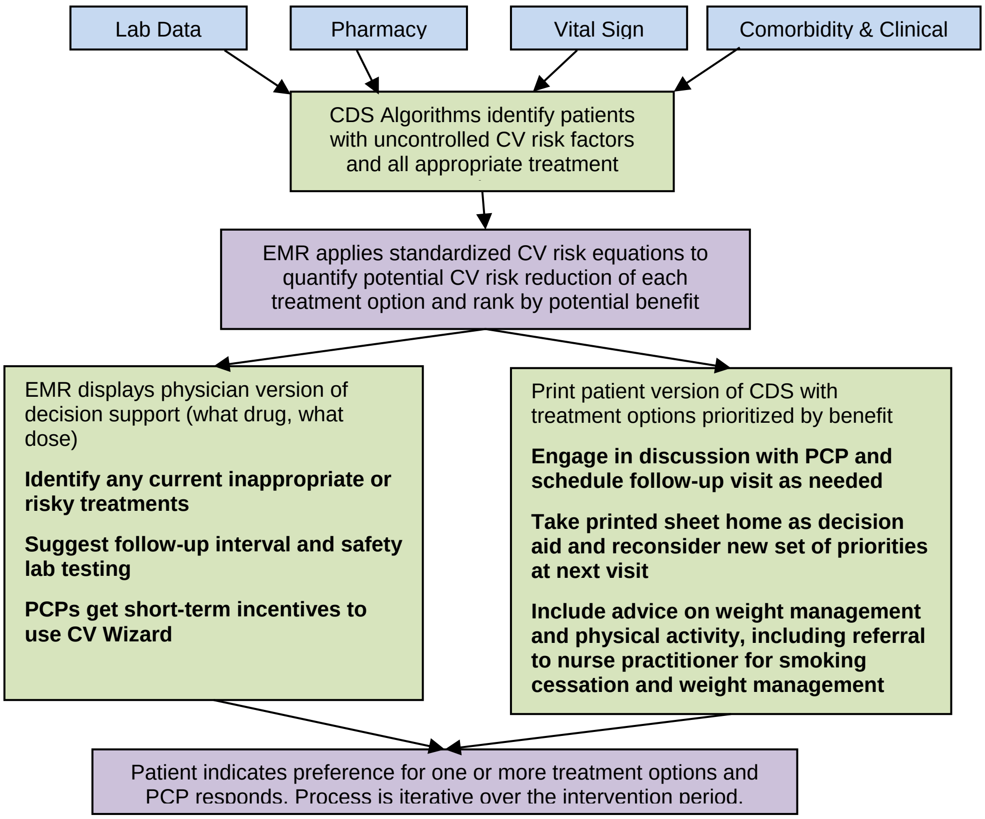

Appendix Figures 1 & 2 show risk-estimation algorithms for BP and aspirin use developed in a previous project (HL102144). The drug effects expected from clinical actions for aspirin use and for all available BP, lipid, and glucose drugs, have been previously quantified (HL102144) and are adjusted downward for patients already on intensive multidrug regimens for BP or glucose control.[70, 71] The previous work on

Figure 3. Prototype of PCP Version of CV Wizard.

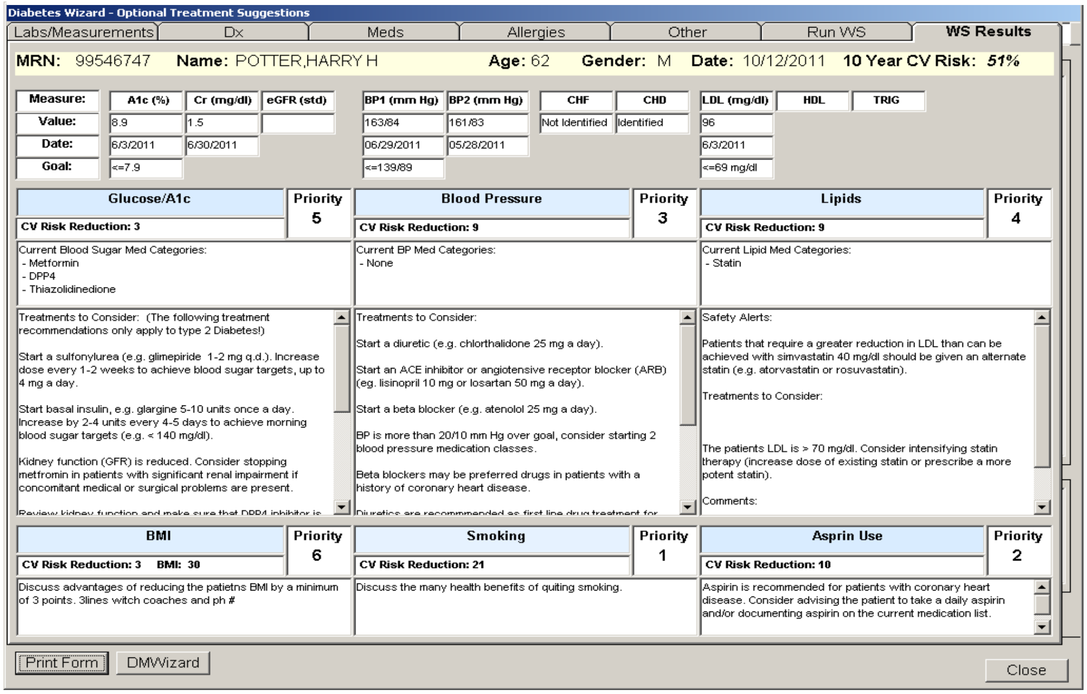

these algorithms makes the proposed project feasible within the proposed timeline and increases the return to the funding agency for building this project on a strong foundation of previous research done by our team.

Step 2. Identify Patients with SMI who May Benefit from SMI Medication Review, and Refer Them to Psychiatric Nurse Care Manager. The CDS algorithms identify patients who take selected medications for SMI and have gained $\geq$7% of their body weight in the previous 12 months or have a BMI $\geq$35. The PCP, psychiatric nurse care manager, and treating psychiatrist (if in the same medical group) also receive the CDS information related to SMI medications and other CV risk factors. The PCP is prompted to inform the patient that a discussion of SMI medications with the nurse care manager and the treating psychiatrist may be indicated, and a referral to the nurse care manager is made. The nurse care manager facilitates communication among the patient, PCP, and behavioral health provider and, guided by treatment algorithms specific to bipolar disorder, schizophrenia, and schizophrenia, convey concerns about the SMI medication and possible alternatives to the treating psychiatrist. **PCPs will not recommend changes in SMI medications.**

Step 3: Identify Available Treatment Options for Each Uncontrolled Modifiable CV Risk Factor: A second set of Web-based algorithms (example prototypes in **Appendix Figures 1 and 2**) identify evidence-based treatment options to address each uncontrolled CV risk factor. These algorithms have been tested in two previous research projects and are maintained on the Wizard Web service and updated (and revalidated) as needed to reflect changes in recommended treatments based on modifications in guidelines, evidence, and/or changes in FDA-indicated uses of certain drugs or classes of drugs.

Pharmacologic treatment recommendations in CV Wizard are directed at CV risk factor control and based on (a) all current prescriptions for BP, lipid, and glucose-control medications and aspirin, smoking cessation medications, and obesogenic SMI medications, (b) available information on the patient's renal or liver function, creatine kinase level, and established diagnoses of diabetes, congestive heart failure, or coronary heart disease, (c) personally assigned BP, A1c, and lipid goals based on comorbidity and other factors (risk of hypoglycemia, intensity of current glucose, BP or lipid regimen), and (d) medication allergies listed in the EMR. Medication recommendations are specific (what drug, what dose) and are based on evidence-based clinical protocols and decision rules developed by our team in previous research projects. These protocols and rules are derived from the evidence-based national and regional (Institute for Clinical Systems Improvement) clinical guidelines for glucose, BP, and lipid control, aspirin use, smoking cessation and obesity management.[72-80]

If inappropriate, CV-related pharmacotherapy or risky prescribing events (including those related to SMI medications, such as concomitant use of lithium carbonate and a diuretic) are identified, CV Wizard will flag them and suggest alternative clinical actions (in the example, substitute a safer BP-lowering agent for the diuretic). A list of prescribing events and a protocol of pharmacotherapy intensification advice given for several hundred clinical scenarios has been tested in a previous project and reported by our group,[81-83] and we will develop a similar list of risky prescribing events specific to the SMI population in phase 1 of this study.

Lifestyle treatment recommendations are also provided, when appropriate. If lifestyle interventions are indicated for smoking or body mass index (BMI) management, and the patient indicates interest, the PCP uses a link in the CV Wizard to refer the patient directly to existing services at each site. Research shows that standard interventions for weight loss or smoking cessation are not often offered to patients with SMI, but that when they are, they can successfully make significant life changes.[38, 84] In this project, the nurse care manager will receive training in weight management and tobacco cessation and reinforce them as indicated.

Step 4: Present Prioritized CV Risk Reduction Options and SMI Treatment Review Recommendations to PCPs and Patients. Prioritized CDS is provided to PCPs and patients using tested interface formats and a sequence of office staff steps successfully implemented in previous studies. Participating PCPs and all rooming nurses in intervention clinics are trained to use the PCP and patient interfaces of CV Wizard, and will take the following steps at each visit: (i) After entering vital sign data, CV Wizard automatically presents the interface screen to the nurse (with no prompts or trigger needed). The rooming nurse prints the **patient and PCP versions** of the CDS sheet. (ii) If a patient's mental and physical status appears stable, the nurse hands the patient sheet (**Figure 4**) and says, "This sheet shows how you can reduce your danger of a stroke or heart attack. Circle any of the things you might want to work on, and let the doctor know during your visit today." (iii) A printed version of the PCP CDS (**Figure 3**) is placed in the basket outside the exam room for rapid review by the PCP before entering the exam room. If preferred, the PCP CDS interface is displayed on the EMR screen

when the PCP enters the room and can be viewed by pressing a button on the EMR navigator bar. (iv) The PCP assesses patient preference for the prioritized CV risk-reduction options. If the patient wants to act on one or more, the PCP can address them immediately or schedule another visit.

PCP Version. CDS provided to the PCP is very specific and identifies and prioritizes treatment options that will, if implemented successfully, reduce CV risk **(Figure 3).** If CV risk-reduction opportunities are identified, the CDS algorithms will specify either initiation or titration of specific drugs based on current medications, distance from goal, and other clinical and comorbidity considerations outlined above. The new interface developed for this project will include a special section reserved for SMI medication review, if indicated. The PCP views this interface before entering the exam room and uses it as a powerful visit-planning tool.

Patient Version. The patient version has a visual display of risk derived from Framingham risk equations and other risk-estimation tools (**Figure 4**).[68, 85, 86] This has been used on a pilot basis in earlier studies, has been well-received by patients with and without SMI, does not heavily depend on numeracy, and has been shown to be strongly motivational.[87-89,90, 91] One display panel (not shown) is reserved to indicate whether a review of SMI medications may be beneficial. If so, the text will read “You may benefit from reviewing your psychiatric medication(s) with your psychiatrist. A nurse care manager will communicate with you in the next 2 weeks to discuss this.”

Figure 4. Prototype Patient Version of CV Wizard.

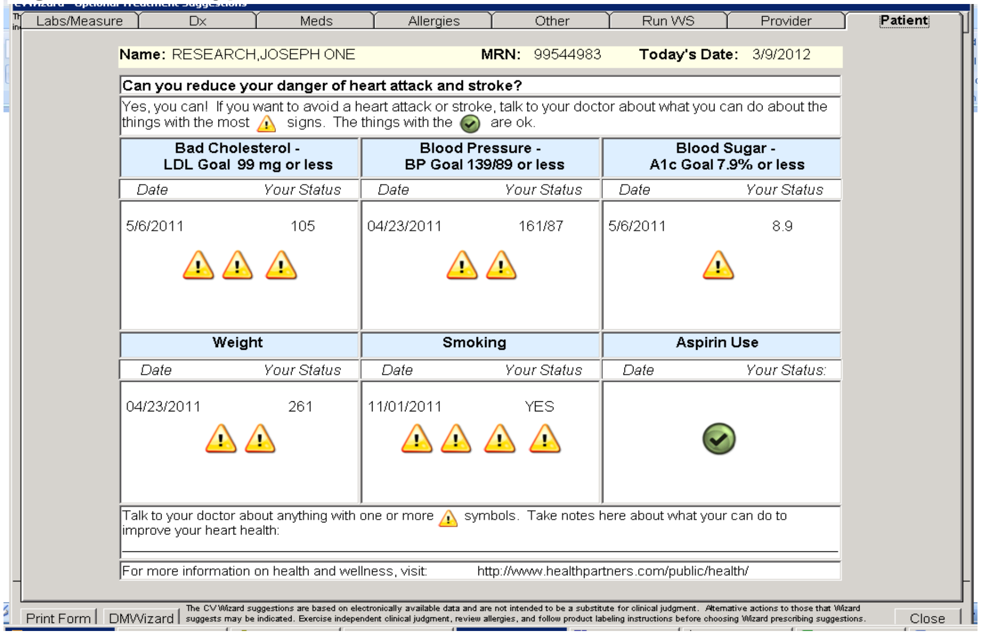

Incorporating Patient Preference.
A key design feature of the patient interface is the presentation of several prioritized treatment options directly to the patient for consideration. Patient preference is elicited simply by asking the patient if they are interested in any of the identified treatment options. Because patient readiness to take health-related actions varies with specific actions, offering several options improves the chance that a given patient may be interested in addressing at least one of them. Moreover, patient readiness to act is a key predictor of subsequent adherence and success of treatment, as we and others have shown.[92-97]

Step 5: Nurse-Supported Medication Switch Protocol. In the subset of patients for whom the Wizard recommends a change in antipsychotic or mood stabilizer medication, a copy of the PCP and patient interfaces are sent automatically to the psychiatric nurse care manager. The psychiatric nurse care manager will (under supervision by and with the approval of the prescribing psychiatrist and consent of the patient) implement a step-wise medication transition protocol (developed in Phase 1) involving weekly standardized telephone assessments of potential adverse effects or indications of symptom breakthrough. In the subset of patients identified for psychiatric medication review, special attention will be given to SMI medication adherence and persistence, trajectory of CV risk factors, and frequency of mental health hospitalizations. However, substantial literature has documented the safety and effectiveness of psychiatric nurse care managers in the care of patients with depression and SMI.[98-100]

Step 6: Strategies to Ensure High Use of CV Wizard. We will implement three strategies to ensure high use rates for CV Wizard: (A) The Wizard screen includes a visit-resolution form that invites the PCP to click, before closing the encounter, one of three boxes: (1) action taken based on Wizard recommendations, (2) other CV risk-reduction action taken, or (3) no CV risk-reduction action taken. If box (3) is clicked, another box must be clicked to indicate why no action was taken. This tool serves multiple purposes: it incentivizes the PCP to take action to avoid additional clicks, allows us to track the percentage of visits each intervention group PCP is using Wizard, and gives feedback to PCPs and clinic leaders on comparative use of CV Wizard. (B)

Benchmarking feedback is given to PCPs on their use of CV Wizard, and medical group leaders at each site will communicate this feedback to each PCP by email every month. (C) Intervention clinics will modify nurse rooming procedures—a change authorized and supported by clinical leaders at each site. (D) The rooming nurses at each clinic will receive one-time compensation of $350 as a group if, in the first 6 months after CV Wizard implementation at their clinic, the CV Wizard interface sheets for the PCP and patient are printed at ≥80% of the visits of patients with SMI. In addition, each site will approve an explicit protocol for psychiatric nurse care manager communication with treating psychiatrists, PCPs, and patients. These strategies are designed to reinforce office nurse, PCP, and psychiatric nurse care manager adherence to study protocols.

Step 7: Interactive Use of the CV Wizard CDS over a Series of Visits. PCPs will use CV Wizard repeatedly at all visits of eligible patients over the intervention period of 18-30 months, depending on site. Pilot data from HealthPartners indicate that adults with SMI who have a PCP (75% do) make an average of ≥5 primary care visits over 24 months, and those who can be engaged in CV risk-reduction activities may visit even more often.[78, 101] We and others have shown that there is substantial relapse to uncontrolled risk-factor status (BP, lipids, smoking, aspirin use, and glucose control) over time. In diabetes patients, for example, about 30% of those with adequate glucose control relapse to elevated levels of glucose within 1 year.[101] CV Wizard identifies and responds to any relapse in CV risk-factor control at each visit. Moreover, with respect to tobacco cessation or weight management, patient preferences are incorporated explicitly into the patient interface. For most patients with SMI in the study, PCPs will have multiple opportunities to consider and implement clinical actions to reduce CV risk over a series of visits during the intervention period.

Implementation of CV Wizard Intervention

In **Phase 1**, we will work closely with clinical leaders, clinic managers, programmers, and IT experts at each site to revise CV Wizard and refine the intervention protocol. Once the initial programming is complete, we will create a series of “dummy” patients with varying CV risk profiles (eg, elevated BP, smoking, candidates for change in psychotropic medications). Clinical investigators (Rossom, Druss, Luepker, and O’Connor) will test the functionality of our algorithms and assess the appropriateness of the CDS. After several rounds of testing and modifications, we will recruit three HealthPartners clinics not included in the study and pilot-test CV Wizard at each clinic for 4 weeks. Pilot-test PCPs and nurses will be asked to complete online surveys and provide within-Wizard feedback on their experience with the CDS tool, including the clinical plausibility and utility of prompts and their impact on clinic workflow. After pilot testing of CV Wizard, the project will enter Phase 2.

Following block randomization of clinics at the start of **Phase 2**, we will train intervention clinics to use CV Wizard using strategies similar to those routinely used to introduce any new EMR functionality. These include group and individual meetings with all PCPs, rooming nurses, and other primary care clinic staff, and email reminders with links to a PowerPoint presentation demonstrating tool use. Training sessions via webinar will also be provided for psychiatrists at each site. Psychiatric nurse care managers will be trained separately by webinar in three sequential 4-hour trainings sessions that include site-specific methods to communicate with within- and outside-group treating psychiatrists. Training will be complete and CV Wizard fully implemented at all intervention clinics within 60 days of randomized group assignment of clinics at each site. Following implementation, all intervention clinic staff will receive monthly email feedback on use of CV Wizard. Surveys of all providers at intervention and control clinics to assess SMI-related knowledge and beliefs will be conducted at baseline and 12 months after clinic randomization. PCP survey data will be used to identify factors that may mediate the impact of CV Wizard.

In addition, we will conduct surveys with a subset of subjects referred to the nurse care manager for medication review to determine: 1) satisfaction with the intervention; 2) their decision to continue current medications or switch medications, and why; 3) whether they felt included in medication and care decisions; 4) whether they perceived benefit from the intervention; 5) level of activity and quality of their diet; 6) what they identify as the most significant contributors to their weight gain; 7) whether they experienced adverse outcomes (eg, psychiatric destabilization and/or hospitalization); 8) perceived barriers in obtaining primary and mental health care; 9) suggestions for improving the EMR patient interface or the care manager intervention.

Measurement of Dependent Variables

CV Risk (Hypothesis 1). The Framingham 10-year CV Disease Risk Equation will be used to estimate the 10-year CV risk for each adult patient with SMI[102] and calculated every time a constituent modifiable risk factor is recorded in the EMR during the intervention period. An index CV risk score will be calculated from data

elements valid at each patient's first primary care visit after randomization of his/her primary care clinic. All patients who meet the study inclusion criteria and have an index modifiable CV risk score >0 will be included in the analytic dataset. At each encounter, we will extract from the EMR the most recent data elements for computing CV risk, looking back over a period appropriate for each risk component (ie, 12 months for A1c, BP, aspirin, smoking, BMI; 48 months for lipids). Risk factors that are unavailable and prevent CV risk calculation will be multiply imputed from a congenial imputation model (see "Missing Data" for details) so that CV risk scores may be calculated for all patients from updated valid and imputed risk factors. The series of CV risk scores will enable person-specific CV risk trajectories to be estimated and compared among patients treated in CV Wizard clinics relative to control clinics. A comparison of model-predicted 12-month post-index CV risk scores demonstrating lower modifiable CV risk in patients in the CV Wizard relative to control clinics will support the primary efficacy of Hypothesis 1.

Modifiable CV Risk factors (Hypothesis 2). Each modifiable CV risk factor recorded in the EMR from those included in the index CV risk estimation through the end of the intervention period will be retained. Person-specific trajectories for each risk factor will be estimated from EMR-recorded risk factors (ie, no imputed values) and compared across clinic-randomized treatment groups.

Obesogenic SMI Medications. Each patient will be classified as having an open prescription for an obesogenic SMI medication at baseline if there was a combination of fill date and days' supply for a defined set of obesogenic atypical antipsychotic or mood stabilizer medications that overlaps with the date of the patient's first primary care visit after randomization of his/her primary care clinic. A comparable 12-month measure will be calculated. The obesogenic agents included in these calculations are valproic acid, olanzapine, clozapine, thioridazine, chlorpromazine, lithium, quetiapine, and risperidone. The proportion of patients with an open prescription for an obesogenic SMI medication will be compared across clinic-randomized treatment groups.

Dependent Variables: Secondary Analyses. A detailed description of dependent variables that will be included in secondary analyses, some of which will pinpoint factors that mediate intervention efficacy is found in **Appendix Table 1**. We postulate that the benefits of CV Wizard will be mediated in part by higher rates of treatment intensification at visits with uncontrolled CV risk factors and will therefore assess (a) BP, glucose, and lipid treatment; and (b) BP, glucose, and lipid treatment intensification at visits with uncontrolled BP, glucose, or lipids. Additional mediating factors include (c) adherence to antipsychotics and mood stabilizers; and (d) number of outpatient and inpatient encounters (total and related to SMI), if CV Wizard increases number of outpatient visits, email visits, emergency department visits, or hospitalizations. CV Wizard might increase the frequency of inappropriate or risky drug prescribing; therefore, we will quantify occurrence of (e) potentially risky prescribing events using methods similar to those we previously published.[81-83] Finally, EMR and claims data will quantify the occurrence and date of (f) major CV events (including fatal and nonfatal heart attack or stroke, total mortality, hospitalized congestive heart failure, and revascularization procedures) so that the occurrence and time to event can be assessed. It is unlikely that the study has power to detect changes in CV events, but we will assess available data in secondary analysis.

Measurement of Independent Variables. (Detailed table found in **Appendix Table 1**)
Primary Predictors. The primary predictor of CV risk trajectories is a binary indicator for whether the clinic in which the patient is seen was randomly assigned to the CV Wizard or control study arm. To test Hypotheses 1 and 2, a second predictor will quantify the time elapsed from the patient's first post-randomization clinic visit, at which time the index CV risk score was calculated, to each point-in-time CV risk score. For Hypothesis 3, a binary indicator will denote whether outcomes were assessed at index or 12 months later.

Patient and Provider Characteristics. Patient and provider characteristics will be documented so we can assess the extent to which results apply to subgroups of patients or whether patient or provider characteristics modify intervention efficacy. Also, clinic randomization may induce random and selection-induced patient covariate imbalance, necessitating adjustment. Patient characteristics from the EMR (for patients) and health plan data sources (for member-patients) include: health plan enrollment and pharmacy coverage dates, demographics, pre-intervention comorbidities (derived from dated ICD-9 codes), vital signs, height, BMI, laboratory values, and prescribed and filled medications. Furthermore, primary care visit dates will link patients and PCPs. We will have complete data for a set of provider characteristics, including age, years since graduation, gender, full-time or part-time status, physician or allied provider (ie, nurse practitioner), specialty board certification status, years with HPMG/KPGA/Essentia, and number and proportion of patients with SMI.

We will also collect data on the percentage of applicable encounters where CV Wizard was printed and/or viewed, and the percentage of encounters with an opened CV Wizard where CV risk factors were addressed.

Analysis Plan.

Hypotheses 1 and 2. H1 and H2 pertain to the efficacy of CV Wizard in improving CV risk and individual risk-factor trajectories. H1 and H2 will be tested using a random coefficients model in which estimated CV risk scores (H1) or factors (H2) will be predicted from clinic-randomized treatment group (WIZARD), time elapsed since index CV risk score (YEAR), and treatment-by-time interaction. The most basic form of the H1 and H2 models will be:

$$\text{CV risk}_{kjit} = \gamma_{0000} + \gamma_{1000}\text{WIZARD}_k + \gamma_{0001}\text{YEAR}_t + \gamma_{1001}\text{WIZARD}_k{*}\text{YEAR}_t + \gamma_{2000}\text{KPGA}_k + \gamma_{3000}\text{EH}_k + [w_{k000} + v_{kj00} + u_{kji0} + u_{kji1}{*}\text{YEAR}_t + e_{kjit}],$$

where there are fixed effects for MHRN site; CV risk varies randomly across clinics (w_{k000}), providers (v_{kjoo}), patients (u_{kji0}), and time (e_{kjit}), and the relationship between time and CV risk varies randomly across patients (u_{kji1}*YEAR_t). These outcomes are expected to be normally or binomially distributed, although the suitability of alternate distributions (eg, negative binomial) and link functions (eg, log) will be assessed should they depart from their expected distributions. YEAR will be coded on a linear scale [(days since index)/365.25] so 0=index and 1=12 months, which should be sufficient to model change in CV risk over time. Quadratic or cubic terms will be tested and added if CV risk trajectories are nonlinear. Parameter γ_{0001} is expected to be near zero, indicating that, among control patients, CV risk did not change over time. Parameter γ_{1001} quantifies the change in CV risk expected at 12 months (when YEAR=1) among WIZARD versus control patients. If γ_{1001} is significant, a planned contrast will test the difference in CV risk outcomes in WIZARD relative to control patients at 12 months. H1 and H2 will be supported if γ_{1001} is significant and negative, and the planned contrast confirms lower predicted CV risk scores at 12 months in WIZARD versus control patients.

Hypothesis 3. The H3 analyses will predict the presence of an open prescription for an obesogenic SMI medication in patients with a 7% or more weight gain in the previous 12 months or whose most recent BMI >= 35 kg/m^2. Each patient will be included in the analysis for the time points at which they met either criterion. The likelihood of an obesogenic SMI medication will be predicted from clinic-randomized treatment group (WIZARD), assessment point (12m), and the treatment by assessment point interaction. The H3 model,

$$\text{Prob(obesogenic SMI medication)}_{kjit} = \gamma_{0000} + \gamma_{1000}\text{WIZARD}_k + \gamma_{0001}\text{12m}_t + \gamma_{1001}\text{WIZARD}_k{*}\text{12m}_t + \gamma_{2000}\text{KPGA}_k + \gamma_{3000}\text{EH}_k + \gamma_{0010}\text{BMI}{>}35_i + [w_{k000} + v_{kj00} + u_{kji0} + e_{kjit}],$$

will specify a binomial error distribution and logit link function, and account for whether the patient BMI >= 35 kg/m^2, in addition to the H1 and H2 model parameters. Due to clinic randomization, the likelihood of a patient taking one of these medications should be similar across treatment groups at index (non-significant γ_{1000}) and, without intervention, be stable over time (nonsignificant γ_{0001}). Parameter γ_{1001} is expected to be significant and negative, implying that CV Wizard patients are less likely than control patients to have an open prescription for an obesogenic SMI medication 12 months after an index visit.

Sample size justification. We conducted a power analysis with PinT software[103] to estimate the standard error of random parameter γ_{1001} in the H1 and H2 models so the regression parameter representing the minimum detectable standardized effect (MDSE) could be calculated. The H1 and H2 data will consist of about four CV risk observations per person per year (using Table 1 preliminary data) in each of about 2,250 patients with SMI receiving primary care in one of 52 randomly assigned clinics. The correlated sample size of N≈2,250 was reduced to an equivalent independent sample size by dividing N by the design effect introduced by patients being clustered within clinics ($N_{eff} = N / [1 + (n_{clus} - 1)\rho]$). The effective sample size Neff≈1,219 to 1,582 (when ρ=.01-.02), expected number of observations per person, and assumptions regarding the within-person covariance matrix, the residual variance in CV risk (97.5% residual), and the random covariance matrix (proportion of residual variance in CV risk at person level, residual slope variance, intercept-slope covariance) formed the basis of power analyses for each CV risk factor. We assumed that the analytic model would explain 2.5% of the variance in each outcome, and the proportion of variance at person and time levels of the model were based on preliminary data (eg, person-level ICC_{DBP} = .35 through ICC_{weight} = .88). The estimated standard errors of γ_{1001} were .028 to .039, which implies MDSEs of d≈.047-.068 (power=.80, α_2=.05). These estimates were consistent in a range of assumptions of patient sample size, person-level residual variance, slope variance, and intercept-slope covariance. Therefore, we anticipate the H1 and H2 analyses will be sufficiently powered to detect small differences (d<.10) in CV risk and CV risk factors among CV Wizard versus control patients 12 months after exposure to the intervention.

For H3, we anticipate that 50% of adult patients with SMI will have experienced a 7% weight gain over 12 months or have BMI >= 35 kg/m^2 at any point. In our pilot, about 70% of patients with SMI were on an antipsychotic or a mood stabilizer, and about 40% of them were obesogenic. We expect, then, that without intervention, about 70%*40%≈30% of patients will have an open prescription for an obesogenic SMI medication at one point. Assuming that the clinic-level ICC_{weight} = .01 -.02 among the N≈2250*.50≈1,125 patients eligible for inclusion at each point, Neff = 796-933. Comparison of CV Wizard to control patients will be powered to detect an absolute 8.0%-8.7% reduction (eg, 21.3%-22.0% CV Wizard vs. 30% control) in the proportion of patients with an open prescription for an obesogenic medication at 12 months.

Missing data. Because all data elements will be drawn from EMR and health plan records, they will be high-quality and available for virtually all patients. Missing data will be rare and can be assumed to be missing at random. The absence of measured risk factors necessary for computing 10-year CV risk will result from its lack of measurement. One concern regarding absent risk-factor measures (eg, LDL values) is that tests are more likely to be performed for patients with known or suspected medical conditions, creating an upward bias in CV risk estimates. There was no evidence of such bias in the central tendency or dispersion of the risk-factor distributions in the preliminary data. However, should the CV Wizard increase CV risk factor monitoring, we may conduct a secondary analysis on data from CV Wizard and control patients, propensity-matched on characteristics that assess primary care involvement primary care (eg, numbers of visits, tests, medications). Because 10-year CV risk will be calculated from risk factors measured in variable-specific timeframes, lack of measurement of a single risk factor could prevent CV risk calculation. To prevent this, we will multiply impute missing risk factors necessary for computing CV risk from all variables in the primary analytic model,[104, 105] including random effects (ie, congenial imputation model with random effects[106]), and other risk factors and patient characteristics that may improve the precision of the imputations using fully conditional specification.

Secondary analyses. The primary analytic model is sufficiently flexible to accommodate non-Gaussian data by specifying alternate error distributions and link functions. As such, secondary efficacy outcomes (eg, medication adherence, safety outcomes, risky prescribing events) will be analyzed using comparable approaches as for the primary analyses, with distributional accommodations as needed. Sub-analyses using the same analytic approach but limited to a) intervention patients whose providers acted based on CV Wizard recommendations or took other CV action, and b) propensity-matched patients, will estimate the maximum effect of the CV Wizard intervention. Medication will be assessed via a product of coefficients approach[107, 108] using comparable mixed models to estimate the strength of relationships among predictors, mediators, and outcomes. The strength of indirect effects will be calculated from model-derived coefficients and its significance tested by constructing asymmetric 95% confidence intervals[109, 110] to determine if its limits include 0.

Organization of Project. The organizational chart and project timeline are provided in the budget justification section of this grant. As PI, Dr. Rossom will lead weekly meetings with the research team (Drs. Owen-Smith, Waring, O'Connor, and Crain, EMR programmers from each site, Web programmers, and project managers) to ensure that all necessary tasks are completed in a timely fashion and strictly according to study protocol. In addition, Dr. Rossom will conduct data meetings with the EMR and Web programmers weekly in the first 30 months of the project and biweekly thereafter to deal with operational issues related to development and implementation of the intervention tool (in Phase 1) and with data and analysis issues throughout the project. Additional clinicians and consultants, including Drs. Trangle, Druss, Luepker, and O'Connor will join the research team meetings to give clinical input and other guidance as needed at certain stages of the project.

In **Phase 1** (Months 0-18), we will develop, pilot, and revise the EMR-based CDS intervention, including the EMR-based algorithms that identify and extract data in real time and the Web service algorithms that process this information and return it to the EMR for retention and display. In **Phase 2** (Months 18 to 48), clinics are randomly assigned and providers trained to use intervention tools in a staggered implementation design to allow each site to learn lessons from the site(s) implementing the intervention previous and increase efficiency. To that end, HealthPartners clinics will implement CV Wizard in month 18, Kaiser Georgia in month 24, and Essentia in month 30. The impact of the intervention on clinical actions, the possible maintenance or decay of intervention effects, and post-intervention PCP satisfaction are monitored over the entirety of the intervention period for a mean of 24 months study-wide. **Phase 3** (Months 48 to 60) involves final data collection, including all clinical outcomes, data analysis, reporting of key study results, and implementation of the intervention in non-intervention clinics, if care partners request (as expected). Throughout the study, data will be analyzed as

they become available, and preliminary reports on the study's conceptual models, intervention strategies, and preliminary results will be reported at meetings and in peer-reviewed articles.

Strengths and Limitations of the Study

A few limitations to this proposal should be noted: (1) The completeness of EMR-derived data is a challenge. However, study subjects have a single EMR used by all providers at each site, and most receive nearly all their care at that site. We have worked extensively with EMR-derived data in patients with depression, diabetes, and heart disease in previous research, and this experience informs our approach to missing data and accurate identification of those with SMI. (2) Although the patient population is large and diverse, it does not include the uninsured; some may argue that this may exclude patients with more severe SMI who are more likely to be uninsured or inconsistently insured. (3) No CV risk-estimation equation is perfectly suited to the needs of patients with SMI, and the Framingham equations may overestimate index risk and underestimate risk reduction catalyzed by CV Wizard because it will miss actions such as use of aspirin and improved glucose control. However, we compensate for this by quantifying changes in key CV risk factors separately in Hypothesis 2, which is a very highly powered analysis, using the UKPDS risk equation for changes in A1c control and the USPSTF aspirin algorithms, which rely on the Framingham 10-year CVD Risk Equation.[111, 112] (4) Including preferences of patients with SMI may be a challenge. However, we have substantial experience in this area,[92, 113,94,114] and previous studies suggest that shared decision making can improve clinical outcomes in patients with chronic conditions.[115] (5) The effectiveness of our recommended lifestyle interventions remains uncertain. Some regard lifestyle interventions as ineffective, but a growing body of evidence suggests commonly available lifestyle interventions and support may positively affect weight, physical activity, and tobacco use in adults, including those with SMI.[116-120]

These potential limitations should be weighed against the strengths of this ambitious, timely, and innovative project. Our health care system invests billions of dollars each year in EMR systems that have failed to deliver clinical benefits to patients with SMI in outpatient settings.[121-126] In previous NIH-funded projects, we have developed an EMR-based, Web-supported CDS system proven to improve BP and glucose control in type 2 diabetes patients in a randomized trial. Pilot data from DK068314 indicate improvement in CV risk factors among a small subset of patients with SMI enrolled in the intervention arm relative to those enrolled in the control arm. Our track record supports our ability to successfully conduct this ambitious and important project. Key elements of the intervention strategy have already been successfully used, enabling us to immediately focus on substantial enhancements to extend the model to our population with SMI, develop maximally effective psychiatric nurse care manager interventions to help patients with SMI switch SMI medications under supervision of the treating psychiatrist when appropriate, including the development of switching algorithms to guide providers, and incorporate patient preference in the process.

Dissemination and Future Applications

The Dissemination Team will meet regularly in the second half of the grant, and will be tasked with ensuring widespread dissemination of our findings. There are many venues readily available to us for useful dissemination; for example, our consultants, Drs. Druss, Luepker, and Trangle, are national leaders in mental health and cardiology and will help ensure regional and national dissemination of results. Additionally, MHRN is an active network whose member organizations have regional and national connections to organizations and collaboratives across the country, including the National Alliance on Mental Illness, the Depression and Bipolar Support Alliance, and the Institute for Clinical Systems Improvement, and these connections will be utilized to widely disseminate our results and spread of our intervention, if successful. In addition to these efforts, we will make good use of the peer-reviewed literature for more traditional dissemination of scientific information. Dr. Rossom will lead a Publications Committee as part of the Dissemination Team, which will develop and facilitate the development of many manuscripts keyed to the specific aims so as to ensure prompt and complete publication of as much important knowledge as possible.

Regarding future applications, the intervention strategy developed here can be extended to other mental health domains and care-delivery venues. Moreover, the Web-based CDS tools could be easily adapted for other purposes, including mapping quality of care at the provider or clinic level, improving coordination of medical care of patients with SMI, providing a framework for "patient-direct" applications delivered through the Internet, and using the CV Wizard in simulation mode to train a wide range of providers to deliver care that is simultaneously personalized and standardized to those with SMI.

1. Parks J, Svendsen D, Singer P, Foti M. Morbidity and Mortality in people with serious mental illness. Alexandria, VA2006. Available from: http://www.nasmhpd.org/general_files/publications/med_directors_pubs/Technical%20Report%20on%20Morbidity%20and%20Mortaility%20-%20Final%2011-06.pdf.
2. Weiner M, Warren L, Fiedorowicz JG. Cardiovascular morbidity and mortality in bipolar disorder. Ann Clin Psychiatry. 2011;23(1):40-7. Epub 2011/02/15. doi: acp_2301f [pii]. PubMed PMID: 21318195; PubMed Central PMCID: PMC3190964.
3. Osborn DP, Levy G, Nazareth I, Petersen I, Islam A, King MB. Relative risk of cardiovascular and cancer mortality in people with severe mental illness from the United Kingdom's General Practice Rsearch Database. Arch Gen Psychiatry. 2007;64(2):242-9. Epub 2007/02/07. doi: 10.1001/archpsyc.64.2.242. PubMed PMID: 17283292.
4. De Hert M, Dekker JM, Wood D, Kahl KG, Holt RI, Moller HJ. Cardiovascular disease and diabetes in people with severe mental illness position statement from the European Psychiatric Association (EPA), supported by the European Association for the Study of Diabetes (EASD) and the European Society of Cardiology (ESC). Eur Psychiatry. 2009;24(6):412-24. Epub 2009/08/18. doi: 10.1016/j.eurpsy.2009.01.005. PubMed PMID: 19682863.
5. O'Connor PJ, Crain AL, Rush WA, Sperl-Hillen JM, Gutenkauf JJ, Duncan JE. Impact of an electronic medical record on diabetes quality of care. Ann Fam Med. 2005;3(4):300-6. Epub 2005/07/28. doi: 3/4/300 [pii] 10.1370/afm.327. PubMed PMID: 16046561; PubMed Central PMCID: PMC1466905.
6. Osby U, Correia N, Brandt L, Ekbom A, Sparen P. Mortality and causes of death in schizophrenia in Stockholm county, Sweden. Schizophr Res. 2000;45(1-2):21-8. Epub 2000/09/09. doi: S0920-9964(99)00191-7 [pii]. PubMed PMID: 10978869.
7. Hennekens CH, Hennekens AR, Hollar D, Casey DE. Schizophrenia and increased risks of cardiovascular disease. Am Heart J. 2005;150(6):1115-21. Epub 2005/12/13. doi: S0002-8703(05)00125-0 [pii] 10.1016/j.ahj.2005.02.007. PubMed PMID: 16338246.
8. Dixon L, Postrado L, Delahanty J, Fischer PJ, Lehman A. The association of medical comorbidity in schizophrenia with poor physical and mental health. J Nerv Ment Dis. 1999;187(8):496-502. Epub 1999/08/27. PubMed PMID: 10463067.
9. Davidson S, Judd F, Jolley D, Hocking B, Thompson S, Hyland B. Cardiovascular risk factors for people with mental illness. Aust N Z J Psychiatry. 2001;35(2):196-202. Epub 2001/04/04. doi: anp877 [pii]. PubMed PMID: 11284901.
10. Druss BG, Bradford WD, Rosenheck RA, Radford MJ, Krumholz HM. Quality of medical care and excess mortality in older patients with mental disorders. Arch Gen Psychiatry. 2001;58(6):565-72. Epub 2001/06/29. doi: yoa20211 [pii]. PubMed PMID: 11386985.
11. Trangle M, Gary M, Paul G, Christensen R. Minnesota 10 by 10. Reducing morbidity and mortality in people with serious mental illnesses. Minn Med. 2010;93(6):38-41. Epub 2010/09/11. PubMed PMID: 20827954.
12. Goff DC, Sullivan LM, McEvoy JP, Meyer JM, Nasrallah HA, Daumit GL, et al. A comparison of ten-year cardiac risk estimates in schizophrenia patients from the CATIE study and matched controls. Schizophr Res. 2005;80(1):45-53. Epub 2005/10/04. doi: 10.1016/j.schres.2005.08.010. PubMed PMID: 16198088.
13. Newcomer JW. Medical risk in patients with bipolar disorder and schizophrenia. J Clin Psychiatry. 2006;67 Suppl 9:25-30; discussion 6-42. Epub 2006/09/13. PubMed PMID: 16965186.
14. Kilbourne AM, Cornelius JR, Han X, Pincus HA, Shad M, Salloum I, et al. Burden of general medical conditions among individuals with bipolar disorder. Bipolar Disord. 2004;6(5):368-73. Epub 2004/09/24. doi: 10.1111/j.1399-5618.2004.00138.x. PubMed PMID: 15383128.
15. Birkenaes AB, Opjordsmoen S, Brunborg C, Engh JA, Jonsdottir H, Ringen PA, et al. The level of cardiovascular risk factors in bipolar disorder equals that of schizophrenia: a comparative study. J Clin Psychiatry. 2007;68(6):917-23. Epub 2007/06/27. PubMed PMID: 17592917.
16. Singer PWM, L.H. Modifiable risk factors: environmental, individual behaviors and lifestyle. Presentation. May 2006.
17. Wong CK, Tang EW, Herbison P, Birmingham B, Barclay L, Fu SY. Pre-existent depression in the 2 weeks before an acute coronary syndrome can be associated with delayed presentation of the heart attack. QJM. 2008;101(2):137-44. Epub 2008/01/12. doi: 10.1093/qjmed/hcm153. PubMed PMID: 18187481.

18. Druss BG. Improving medical care for persons with serious mental illness: challenges and solutions. J Clin Psychiatry. 2007;68 Suppl 4:40-4. Epub 2007/08/19. PubMed PMID: 17539699.
19. Laursen TM, Munk-Olsen T, Agerbo E, Gasse C, Mortensen PB. Somatic hospital contacts, invasive cardiac procedures, and mortality from heart disease in patients with severe mental disorder. Arch Gen Psychiatry. 2009;66(7):713-20. Epub 2009/07/08. doi: 10.1001/archgenpsychiatry.2009.61. PubMed PMID: 19581562.
20. Mackin P, Bishop DR, Watkinson HM. A prospective study of monitoring practices for metabolic disease in antipsychotic-treated community psychiatric patients. BMC Psychiatry. 2007;7:28. Epub 2007/06/27. doi: 10.1186/1471-244X-7-28. PubMed PMID: 17592636; PubMed Central PMCID: PMC1913512.
21. Shah SU, White A, White S, Littler WA. Heart and mind: (1) relationship between cardiovascular and psychiatric conditions. Postgrad Med J. 2004;80(950):683-9. Epub 2004/12/08. doi: 80/950/683 [pii] 10.1136/pgmj.2003.014662. PubMed PMID: 15579605; PubMed Central PMCID: PMC1743159.
22. Sowden GL, Huffman JC. The impact of mental illness on cardiac outcomes: a review for the cardiologist. Int J Cardiol. 2009;132(1):30-7. Epub 2008/11/14. doi: 10.1016/j.ijcard.2008.10.002. PubMed PMID: 19004512.
23. Taylor V, MacQueen G. Associations between bipolar disorder and metabolic syndrome: A review. J Clin Psychiatry. 2006;67(7):1034-41. Epub 2006/08/08. PubMed PMID: 16889445.
24. Klumpers UM, Boom K, Janssen FM, Tulen JH, Loonen AJ. Cardiovascular risk factors in outpatients with bipolar disorder. Pharmacopsychiatry. 2004;37(5):211-6. Epub 2004/10/09. PubMed PMID: 15470799.
25. Consensus development conference on antipsychotic drugs and obesity and diabetes. Diabetes Care. 2004;27(2):596-601. Epub 2004/01/30. PubMed PMID: 14747245.
26. Druss BG, Bradford DW, Rosenheck RA, Radford MJ, Krumholz HM. Mental disorders and use of cardiovascular procedures after myocardial infarction. JAMA. 2000;283(4):506-11. Epub 2000/02/05. doi: joc90746 [pii]. PubMed PMID: 10659877.
27. Frayne SM, Halanych JH, Miller DR, Wang F, Lin H, Pogach L, et al. Disparities in diabetes care: impact of mental illness. Arch Intern Med. 2005;165(22):2631-8. Epub 2005/12/14. doi: 10.1001/archinte.165.22.2631. PubMed PMID: 16344421.
28. Desai MM, Rosenheck RA, Druss BG, Perlin JB. Mental disorders and quality of diabetes care in the veterans health administration. Am J Psychiatry. 2002;159(9):1584-90. Epub 2002/08/31. PubMed PMID: 12202281.
29. Fagiolini A, Chengappa KN, Soreca I, Chang J. Bipolar disorder and the metabolic syndrome: causal factors, psychiatric outcomes and economic burden. CNS Drugs. 2008;22(8):655-69. Epub 2008/07/08. PubMed PMID: 18601304.
30. Mitchell JE, Mackenzie TB. Cardiac effects of lithium therapy in man: a review. J Clin Psychiatry. 1982;43(2):47-51. Epub 1982/02/01. PubMed PMID: 7056703.
31. Glassman AH, Bigger JT, Jr. Antipsychotic drugs: prolonged QTc interval, torsade de pointes, and sudden death. Am J Psychiatry. 2001;158(11):1774-82. Epub 2001/11/03. PubMed PMID: 11691681.
32. Morriss R, Mohammed FA. Metabolism, lifestyle and bipolar affective disorder. J Psychopharmacol. 2005;19(6 Suppl):94-101. Epub 2005/11/11. doi: 10.1177/0269881105058678. PubMed PMID: 16280342.
33. Angst F, Stassen HH, Clayton PJ, Angst J. Mortality of patients with mood disorders: follow-up over 34-38 years. J Affect Disord. 2002;68(2-3):167-81. Epub 2002/06/14. doi: S0165032701003779 [pii]. PubMed PMID: 12063145.
34. Ryan MC, Thakore JH. Physical consequences of schizophrenia and its treatment: the metabolic syndrome. Life Sci. 2002;71(3):239-57. Epub 2002/05/30. doi: S0024320502016466 [pii]. PubMed PMID: 12034344.
35. Lieberman JA, Stroup TS, McEvoy JP, Swartz MS, Rosenheck RA, Perkins DO, et al. Effectiveness of antipsychotic drugs in patients with chronic schizophrenia. N Engl J Med. 2005;353(12):1209-23. Epub 2005/09/21. doi: NEJMoa051688 [pii]
10.1056/NEJMoa051688. PubMed PMID: 16172203.
36. Elmer PJ, Obarzanek E, Vollmer WM, Simons-Morton D, Stevens VJ, Young DR, et al. Effects of comprehensive lifestyle modification on diet, weight, physical fitness, and blood pressure control: 18-month results of a randomized trial. Ann Intern Med. 2006;144(7):485-95. Epub 2006/04/06. PubMed PMID: 16585662.

37. Whelton PK, Appel LJ, Espeland MA, Applegate WB, Ettinger WH, Jr., Kostis JB, et al. Sodium reduction and weight loss in the treatment of hypertension in older persons: a randomized controlled trial of nonpharmacologic interventions in the elderly (TONE). TONE Collaborative Research Group. JAMA. 1998;279(11):839-46. Epub 1998/03/27. PubMed PMID: 9515998.
38. Daumit GL, Dickerson FB, Wang NY, Dalcin A, Jerome GJ, Anderson CA, et al. A behavioral weight-loss intervention in persons with serious mental illness. N Engl J Med. 2013;368(17):1594-602. Epub 2013/03/23. doi: 10.1056/NEJMoa1214530. PubMed PMID: 23517118.
39. Pylvanen V, Knip M, Pakarinen A, Kotila M, Turkka J, Isojarvi JI. Serum insulin and leptin levels in valproate-associated obesity. Epilepsia. 2002;43(5):514-7. Epub 2002/05/25. PubMed PMID: 12027912.
40. Sachs G, Bowden C, Calabrese JR, Ketter T, Thompson T, White R, et al. Effects of lamotrigine and lithium on body weight during maintenance treatment of bipolar I disorder. Bipolar Disord. 2006;8(2):175-81. Epub 2006/03/18. doi: 10.1111/j.1399-5618.2006.00308.x. PubMed PMID: 16542188.
41. Allison DB, Mentore JL, Heo M, Chandler LP, Cappelleri JC, Infante MC, et al. Antipsychotic-induced weight gain: a comprehensive research synthesis. Am J Psychiatry. 1999;156(11):1686-96. Epub 1999/11/30. PubMed PMID: 10553730.
42. Komossa K, Rummel-Kluge C, Schmid F, Hunger H, Schwarz S, El-Sayeh HG, et al. Aripiprazole versus other atypical antipsychotics for schizophrenia. Cochrane Database Syst Rev. 2009(4):CD006569. Epub 2009/10/13. doi: 10.1002/14651858.CD006569.pub3. PubMed PMID: 19821375.
43. Patel JK, Buckley PF, Woolson S, Hamer RM, McEvoy JP, Perkins DO, et al. Metabolic profiles of second-generation antipsychotics in early psychosis: findings from the CAFE study. Schizophr Res. 2009;111(1-3):9-16. Epub 2009/04/29. doi: 10.1016/j.schres.2009.03.025. PubMed PMID: 19398192.
44. Hemmes M AD, Gerhard G, McCarty C, Nakasato C, Pawloski P, Rukstalis M, Schmelzer J, Yale S, Davis R. PS1-18: A Feasibility Pilot to Determine the Practicality of Using the HMO Research Network to Research the Genetics of Drug-Induced Serious Adverse Events. Clin Med Res. 2011;9(3-4):2.
45. O'Connor P. Opportunities to increase the effectiveness of EHR-based diabetes clinical decision support. Appl Clin Inf. 2011;2:350-4.
46. Sittig DF, Wright A, Osheroff JA, Middleton B, Teich JM, Ash JS, et al. Grand challenges in clinical decision support. J Biomed Inform. 2008;41(2):387-92. Epub 2007/11/22. doi: S1532-0464(07)00104-9 [pii] 10.1016/j.jbi.2007.09.003. PubMed PMID: 18029232; PubMed Central PMCID: PMC2660274.
47. O'Connor PJ, Sperl-Hillen JM, Rush WA, Johnson PE, Amundson GH, Asche SE, et al. Impact of electronic health record clinical decision support on diabetes care: a randomized trial. Ann Fam Med. 2011;9(1):12-21. Epub 2011/01/19. doi: 10.1370/afm.1196. PubMed PMID: 21242556; PubMed Central PMCID: PMC3022040.
48. Leibson C, Owens T, O'Brien P, Waring S, Tangalos E, Hanson V, et al. Use of physician and acute care services by persons with and without Alzheimer's disease: a population-based comparison. J Am Geriatr Soc. 1999;47(7):864-9. Epub 1999/07/15. PubMed PMID: 10404933.
49. O'Bryant SE HJ, Waring SC, Humphreys JD, Schiffer RB. The Relationship Between Cardiovascular Disease and Alzheimer's Disease. Texas Public Health Journal. 2008;60(3):3.
50. O'Bryant SE, Waring SC, Cullum CM, Hall J, Lacritz L, Massman PJ, et al. Staging dementia using Clinical Dementia Rating Scale Sum of Boxes scores: a Texas Alzheimer's research consortium study. Arch Neurol. 2008;65(8):1091-5. Epub 2008/08/13. doi: 10.1001/archneur.65.8.1091. PubMed PMID: 18695059; PubMed Central PMCID: PMC3409562.
51. Rummans TA, Smith GE, Lin SC, Waring SC, Kokmen E. Comorbidity of dementia and psychiatric disorders in older persons. Am J Geriatr Psychiatry. 1997;5(3):261-7. Epub 1997/07/01. PubMed PMID: 9209569.
52. Waring SC, Doody RS, Pavlik VN, Massman PJ, Chan W. Survival among patients with dementia from a large multi-ethnic population. Alzheimer Dis Assoc Disord. 2005;19(4):178-83. Epub 2005/12/06. PubMed PMID: 16327343.
53. Bryant MD, Schoenberg ED, Johnson TV, Goodman M, Owen-Smith A, Master VA. Multimedia version of a standard medical questionnaire improves patient understanding across all literacy levels. J Urol. 2009;182(3):1120-5. Epub 2009/07/25. doi: 10.1016/j.juro.2009.05.027. PubMed PMID: 19625036.
54. Johnson TV, Abbasi A, Ehrlich SS, Kleris RS, Chirumamilla SL, Schoenberg ED, et al. Major depression drives severity of American Urological Association Symptom Index. Urology. 2010;76(6):1317-20. Epub 2010/12/07. doi: 10.1016/j.urology.2010.01.069. PubMed PMID: 21130246.

55. Master VA, Johnson TV, Abbasi A, Ehrlich SS, Kleris RS, Abbasi S, et al. Poorly numerate patients in an inner city hospital misunderstand the American Urological Association symptom score. Urology. 2010;75(1):148-52. Epub 2009/10/13. doi: 10.1016/j.urology.2009.06.060. PubMed PMID: 19819537.
56. Johnson TV, Abbasi A, Ehrlich SS, Kleris RS, Owen-Smith A, Raison CL, et al. IPSS quality of life question: a possible indicator of depression among patients with lower urinary tract symptoms. The Canadian journal of urology. 2012;19(1):6100-4. Epub 2012/02/10. PubMed PMID: 22316511.
57. Filion KB, Steffen LM, Duval S, Jacobs DR, Jr., Blackburn H, Luepker RV. Trends in smoking among adults from 1980 to 2009: the Minnesota heart survey. Am J Public Health. 2012;102(4):705-13. Epub 2011/08/20. doi: 10.2105/AJPH.2011.300162. PubMed PMID: 21852651.
58. Luepker RV, Steffen LM, Jacobs DR, Jr., Zhou X, Blackburn H. Trends in blood pressure and hypertension detection, treatment, and control 1980 to 2009: the Minnesota Heart Survey. Circulation. 2012;126(15):1852-7. Epub 2012/09/11. doi: 10.1161/CIRCULATIONAHA.112.098517. PubMed PMID: 22962433; PubMed Central PMCID: PMC3482957.
59. McGovern PG, Pankow JS, Shahar E, Doliszny KM, Folsom AR, Blackburn H, et al. Recent trends in acute coronary heart disease--mortality, morbidity, medical care, and risk factors. The Minnesota Heart Survey Investigators. N Engl J Med. 1996;334(14):884-90. Epub 1996/04/04. doi: 10.1056/NEJM199604043341403. PubMed PMID: 8596571.
60. Druss BG, Rohrbaugh RM, Levinson CM, Rosenheck RA. Integrated medical care for patients with serious psychiatric illness: a randomized trial. Arch Gen Psychiatry. 2001;58(9):861-8. Epub 2001/09/26. doi: yoa20292 [pii]. PubMed PMID: 11545670.
61. Druss BG, von Esenwein SA. Improving general medical care for persons with mental and addictive disorders: systematic review. Gen Hosp Psychiatry. 2006;28(2):145-53. Epub 2006/03/07. doi: 10.1016/j.genhosppsych.2005.10.006. PubMed PMID: 16516065.
62. Simon GE. Depression in medical patients. J Gen Intern Med. 1988;3(2):208. Epub 1988/03/01. PubMed PMID: 3258635.
63. Simon GE, Katon WJ, Lin EH, Ludman E, VonKorff M, Ciechanowski P, et al. Diabetes complications and depression as predictors of health service costs. Gen Hosp Psychiatry. 2005;27(5):344-51. Epub 2005/09/20. doi: 10.1016/j.genhosppsych.2005.04.008. PubMed PMID: 16168795.
64. Simon GE, Ludman EJ, Bauer MS, Unutzer J, Operskalski B. Long-term effectiveness and cost of a systematic care program for bipolar disorder. Arch Gen Psychiatry. 2006;63(5):500-8. Epub 2006/05/03. doi: 10.1001/archpsyc.63.5.500. PubMed PMID: 16651507.
65. Simon GE, VonKorff M, Barlow W. Health care costs of primary care patients with recognized depression. Arch Gen Psychiatry. 1995;52(10):850-6. Epub 1995/10/01. PubMed PMID: 7575105.
66. Simon GE, VonKorff M, Heiligenstein JH, Revicki DA, Grothaus L, Katon W, et al. Initial antidepressant choice in primary care. Effectiveness and cost of fluoxetine vs tricyclic antidepressants. JAMA. 1996;275(24):1897-902. Epub 1996/06/26. PubMed PMID: 8648870.
67. O'Connor PJ, Desai JR, Butler JC, Kharbanda EO, Sperl-Hillen JM. Current status and future prospects for electronic point-of-care clinical decision support in diabetes care. Current diabetes reports. 2013;13(2):172-6. Epub 2012/12/12. doi: 10.1007/s11892-012-0350-z. PubMed PMID: 23225213; PubMed Central PMCID: PMC3595375.
68. D'Agostino RB, Sr., Vasan RS, Pencina MJ, Wolf PA, Cobain M, Massaro JM, et al. General cardiovascular risk profile for use in primary care: the Framingham Heart Study. Circulation. 2008;117(6):743-53. Epub 2008/01/24. doi: 10.1161/CIRCULATIONAHA.107.699579. PubMed PMID: 18212285.
69. Wilson PW, D'Agostino RB, Levy D, Belanger AM, Silbershatz H, Kannel WB. Prediction of coronary heart disease using risk factor categories. Circulation. 1998;97(18):1837-47. Epub 1998/05/29. PubMed PMID: 9603539.
70. Vijan S, Hayward RA. Treatment of hypertension in type 2 diabetes mellitus: blood pressure goals, choice of agents, and setting priorities in diabetes care. Ann Intern Med. 2003;138(7):593-602. Epub 2003/04/02. doi: 200304010-00018 [pii]. PubMed PMID: 12667032.
71. Bennett WL, Maruthur NM, Singh S, Segal JB, Wilson LM, Chatterjee R, et al. Comparative effectiveness and safety of medications for type 2 diabetes: an update including new drugs and 2-drug combinations. Ann Intern Med. 2011;154(9):602-13. Epub 2011/03/16. doi: 10.7326/0003-4819-154-9-201105030-00336. PubMed PMID: 21403054.

72. ICSI. Diabetes Mellitus in Adults, Type 2; Diagnosis and Management of (Guideline). 2008 [April 18, 2012]. Available from: http://www.icsi.org/guidelines_and_more/gl_os_prot/other_health_care_conditions/diabetes_mellitus__type_2/diabetes_mellitus__type_2__management_of___6.html.

73. O'Connor PJ, Sperl-Hillen, J. Treatment of Type 2 Diabetes. Online Point of Care clinical decision support tool marketed as part of BMJ Point of Care. Concise and practical information on etiology, epidemiology, and clinical management of type 2 diabetes. BMJ. 2008.

74. Third Report of the National Cholesterol Education Program (NCEP) Expert Panel on Detection, Evaluation, and Treatment of High Blood Cholesterol in Adults (Adult Treatment Panel III) final report. Circulation. 2002;106(25):3143-421. Epub 2002/12/18. PubMed PMID: 12485966.

75. Chobanian AV, Bakris GL, Black HR, Cushman WC, Green LA, Izzo JL, Jr., et al. The Seventh Report of the Joint National Committee on Prevention, Detection, Evaluation, and Treatment of High Blood Pressure: the JNC 7 report. JAMA. 2003;289(19):2560-72. Epub 2003/05/16. doi: 10.1001/jama.289.19.2560. PubMed PMID: 12748199.

76. Summary of revisions for the 2009 Clinical Practice Recommendations. Diabetes Care. 2009;32 Suppl 1:S3-5. Epub 2009/01/06. doi: 10.2337/dc09-S003. PubMed PMID: 19118287; PubMed Central PMCID: PMC2613585.

77. ICSI. Chronic Disease Risk Factors, Primary Prevention of (Guideline): ICSI (Institute for Chronic Systems Improvement). 2008.

78. O'Connor PJ, Quiter ES, Rush WA, Wiest M, Meland JT, Ryu S. Impact of hypertension guideline implementation on blood pressure control and drug use in primary care clinics. Jt Comm J Qual Improv. 1999;25(2):68-77. Epub 1999/02/23. PubMed PMID: 10027112.

79. Hypertension Diagnosis and Treatment (Guideline). [Internet]. November 2008 [cited May 29, 2011.]. Available from: http://www.icsi.org/guidelines_and_more/gl_os_prot/cardiovascular/hypertension_4/hypertension_diagnosis_and_treatment__11.html. .

80. 2008 PHS Guideline Update Panel L, and Staff. Treating tobacco use and dependence: 2008 update U.S.Public Health Service Clinical Practice Guideline executive summary. Respir Care. 2008;53(9):6. PubMed Central PMCID: PMC18807274.

81. O'Connor PJ, Sperl-Hillen JM, Johnson PE, Rush WA, Asche SE, Dutta P, et al. Simulated physician learning intervention to improve safety and quality of diabetes care: a randomized trial. Diabetes Care. 2009;32(4):585-90. Epub 2009/01/28. doi: dc08-0944 [pii]
10.2337/dc08-0944. PubMed PMID: 19171723; PubMed Central PMCID: PMC2660457.

82. O'Connor PJ, Sperl-Hillen JM, Johnson PE, Rush WA, Biltz G. Clinical Inertia and Outpatient Medical Errors. In: Henriksen KB, J.; Lewin, D.; Marks, E., editor. Advances in Patient Safety: From Research to Implementation. Rockville, MD: Agency for Healthcare Research and Quality (AHRQ); 2005. p. 293-308.

83. O'Connor PJ, Sperl-Hillen JM, Johnson PE, Rush WA. Identification, Classification, and Frequency of Medical Errors in Outpatient Diabetes Care. In: Henriksen KB, J.; Lewin, D.; Marks, E., editor. Advances in Patient Safety: From Research to Implementation. Rockville, MD2005. p. 369-80.

84. Tsoi DT, Porwal M, Webster AC. Interventions for smoking cessation and reduction in individuals with schizophrenia. Cochrane Database Syst Rev. 2013;2:CD007253. Epub 2013/03/02. doi: 10.1002/14651858.CD007253.pub3. PubMed PMID: 23450574.

85. Wilson PW. Risk scores for prediction of coronary heart disease: an update. Endocrinol Metab Clin North Am. 2009;38(1):33-44. Epub 2009/02/17. doi: 10.1016/j.ecl.2008.11.001. PubMed PMID: 19217511.

86. Wilson PW, Castelli WP, Kannel WB. Coronary risk prediction in adults (the Framingham Heart Study). Am J Cardiol. 1987;59(14):91G-4G. Epub 1987/05/29. PubMed PMID: 3591674.

87. Powell LH, Calvin JE, Jr., Mendes de Leon CF, Richardson D, Grady KL, Flynn KJ, et al. The Heart Failure Adherence and Retention Trial (HART): design and rationale. Am Heart J. 2008;156(3):452-60. Epub 2008/09/02. doi: 10.1016/j.ahj.2008.05.011. PubMed PMID: 18760125; PubMed Central PMCID: PMC3609705.

88. van der Wal MH, Jaarsma T. Adherence in heart failure in the elderly: problem and possible solutions. Int J Cardiol. 2008;125(2):203-8. Epub 2007/11/23. doi: 10.1016/j.ijcard.2007.10.011. PubMed PMID: 18031843.

89. Flynn KJ, Powell LH, Mendes de Leon CF, Munoz R, Eaton CB, Downs DL, et al. Increasing self-management skills in heart failure patients: a pilot study. Congest Heart Fail. 2005;11(6):297-302. Epub 2005/12/07. PubMed PMID: 16330904.
90. Baker DW, Williams MV, Parker RM, Gazmararian JA, Nurss J. Development of a brief test to measure functional health literacy. Patient Educ Couns. 1999;38(1):33-42. Epub 2003/10/08. doi: S0738-3991(98)00116-5 [pii]. PubMed PMID: 14528569.
91. Paulos JA. Innumeracy: Mathematical Illiteracy and Its Consequences. . New York: Hill and Wang; 2001.
92. Boyle RG, O'Connor PJ, Pronk NP, Tan A. Stages of change for physical activity, diet, and smoking among HMO members with chronic conditions. Am J Health Promot. 1998;12(3):170-5. Epub 1997/12/08. PubMed PMID: 10176091.
93. Peterson KA, Hughes M. Readiness to change and clinical success in a diabetes educational program. J Am Board Fam Pract. 2002;15(4):266-71. Epub 2002/08/02. PubMed PMID: 12150458.
94. O'Connor PJ, Asche SE, Crain AL, Rush WA, Whitebird RR, Solberg LI, et al. Is patient readiness to change a predictor of improved glycemic control? Diabetes Care. 2004;27(10):2325-9. Epub 2004/09/29. doi: 27/10/2325 [pii]. PubMed PMID: 15451895.
95. Prochaska JO, Velicer WF, Rossi JS, Goldstein MG, Marcus BH, Rakowski W, et al. Stages of change and decisional balance for 12 problem behaviors. Health Psychol. 1994;13(1):39-46. Epub 1994/01/01. PubMed PMID: 8168470.
96. Ruggiero L, Rossi JS, Prochaska JO, Glasgow RE, de Groot M, Dryfoos JM, et al. Smoking and diabetes: readiness for change and provider advice. Addict Behav. 1999;24(4):573-8. Epub 1999/08/31. doi: S0306-4603(98)00086-0 [pii]. PubMed PMID: 10466853.
97. Ruggiero LP, J.O. Readiness for change: application of the transtheoretical model to diabetes. . Diabetes Spectrum. 1993;6(1):21-60.
98. Katon W, Guico-Pabia CJ. Improving quality of depression care using organized systems of care: a review of the literature. The primary care companion to CNS disorders. 2011;13(1). Epub 2011/07/07. doi: 10.4088/PCC.10r01019blu. PubMed PMID: 21731829; PubMed Central PMCID: PMC3121215.
99. Druss BG, von Esenwein SA, Compton MT, Rask KJ, Zhao L, Parker RM. A randomized trial of medical care management for community mental health settings: the Primary Care Access, Referral, and Evaluation (PCARE) study. Am J Psychiatry. 2010;167(2):151-9. Epub 2009/12/17. doi: 10.1176/appi.ajp.2009.09050691. PubMed PMID: 20008945.
100. Rubenstein LV, Chaney EF, Ober S, Felker B, Sherman SE, Lanto A, et al. Using evidence-based quality improvement methods for translating depression collaborative care research into practice. Families, systems & health : the journal of collaborative family healthcare. 2010;28(2):91-113. Epub 2010/08/11. doi: 10.1037/a0020302. PubMed PMID: 20695669.
101. O'Connor PJ, Sperl-Hillen J. Clinical and public health implications of glycemic relapse in type 2 diabetes. Nat Clin Pract Endocrinol Metab. 2007;3(1):10-1. Epub 2006/12/21. doi: 10.1038/ncpendmet0354. PubMed PMID: 17179924.
102. Lloyd-Jones DM, Wilson PW, Larson MG, Beiser A, Leip EP, D'Agostino RB, et al. Framingham risk score and prediction of lifetime risk for coronary heart disease. Am J Cardiol. 2004;94(1):20-4. Epub 2004/06/29. doi: 10.1016/j.amjcard.2004.03.023. PubMed PMID: 15219502.
103. Snijders TAB, R.J. Standard errors and sample sizes in two-level research. . Journal of Educational Statistics. 1993;18(3):237-60.
104. Little RJAR, D.B. Statistical Anslysis with Missing Data. New York: Wiley.; 1987.
105. Rubin DB. Multiple Imputation for Nonresponse in Surveys. New York: Wiley; 1987.
106. Andridge RR. Quantifying the impact of fixed effects modeling of clusters in multiple imputation for cluster randomized trials. Biom J. 2011;53(1):57-74. Epub 2011/01/25. doi: 10.1002/bimj.201000140. PubMed PMID: 21259309; PubMed Central PMCID: PMC3124925.
107. Sobel ME. Asymptotic intervals for indirect effects in structural equation models. In: Leinhart, editor. Sociological Methodology. San Francisco: Jossey-Bass 1982. p. 290-312.
108. Mackinnon DP, Warsi G, Dwyer JH. A Simulation Study of Mediated Effect Measures. Multivariate Behav Res. 1995;30(1):41. Epub 1995/01/01. doi: 10.1207/s15327906mbr3001_3. PubMed PMID: 20157641; PubMed Central PMCID: PMC2821114.

109. MacKinnon DP, Lockwood CM, Hoffman JM, West SG, Sheets V. A comparison of methods to test mediation and other intervening variable effects. Psychol Methods. 2002;7(1):83-104. Epub 2002/04/04. PubMed PMID: 11928892; PubMed Central PMCID: PMC2819363.

110. MacKinnon DP. Introduction to Statistical Mediation Analysis. New York: Erlbaum; 2008.

111. Stevens RJ, Kothari V, Adler AI, Stratton IM. The UKPDS risk engine: a model for the risk of coronary heart disease in Type II diabetes (UKPDS 56). Clin Sci (Lond). 2001;101(6):671-9. Epub 2001/11/29. PubMed PMID: 11724655.

112. Aspirin for the prevention of cardiovascular disease: U.S. Preventive Services Task Force recommendation statement. Ann Intern Med. 2009;150(6):396-404. Epub 2009/03/19. doi: 150/6/396 [pii]. PubMed PMID: 19293072.

113. O'Connor PJ, Sperl-Hillen J, Johnson PE, Rush WA, Crain AL. Customized feedback to patients and providers failed to improve safety or quality of diabetes care: a randomized trial. Diabetes Care. 2009;32(7):1158-63. Epub 2009/04/16. doi: dc08-2247 [pii]
10.2337/dc08-2247. PubMed PMID: 19366977; PubMed Central PMCID: PMC2699722.

114. O'Connor PJ, Rush WA, Prochaska JO, Pronk NP, Boyle RG. Professional advice and readiness to change behavioral risk factors among members of a managed care organization. Am J Manag Care. 2001;7(2):125-30. Epub 2001/02/24. doi: 651 [pii]. PubMed PMID: 11216330.

115. Greenfield S, Kaplan SH, Ware JE, Jr., Yano EM, Frank HJ. Patients' participation in medical care: effects on blood sugar control and quality of life in diabetes. J Gen Intern Med. 1988;3(5):448-57. Epub 1988/09/01. PubMed PMID: 3049968.

116. Wing RR. Long-term effects of a lifestyle intervention on weight and cardiovascular risk factors in individuals with type 2 diabetes mellitus: four-year results of the Look AHEAD trial. Arch Intern Med. 2010;170(17):1566-75. Epub 2010/09/30. doi: 170/17/1566 [pii]
10.1001/archinternmed.2010.334. PubMed PMID: 20876408; PubMed Central PMCID: PMC3084497.

117. Epstein LH, Wing RR, Penner BC, Kress MJ. Effect of diet and controlled exercise on weight loss in obese children. J Pediatr. 1985;107(3):358-61. Epub 1985/09/01. PubMed PMID: 4032130.

118. Li G, Zhang P, Wang J, Gregg EW, Yang W, Gong Q, et al. The long-term effect of lifestyle interventions to prevent diabetes in the China Da Qing Diabetes Prevention Study: a 20-year follow-up study. Lancet. 2008;371(9626):1783-9. Epub 2008/05/27. doi: S0140-6736(08)60766-7 [pii]
10.1016/S0140-6736(08)60766-7. PubMed PMID: 18502303.

119. Lindstrom J, Ilanne-Parikka P, Peltonen M, Aunola S, Eriksson JG, Hemio K, et al. Sustained reduction in the incidence of type 2 diabetes by lifestyle intervention: follow-up of the Finnish Diabetes Prevention Study. Lancet. 2006;368(9548):1673-9. Epub 2006/11/14. doi: S0140-6736(06)69701-8 [pii]
10.1016/S0140-6736(06)69701-8. PubMed PMID: 17098085.

120. Knowler WC, Fowler SE, Hamman RF, Christophi CA, Hoffman HJ, Brenneman AT, et al. 10-year follow-up of diabetes incidence and weight loss in the Diabetes Prevention Program Outcomes Study. Lancet. 2009;374(9702):1677-86. Epub 2009/11/03. doi: S0140-6736(09)61457-4 [pii]
10.1016/S0140-6736(09)61457-4. PubMed PMID: 19878986; PubMed Central PMCID: PMC3135022.

121. O'Connor PJ. Electronic medical records and diabetes care improvement: Are we waiting for Godot? Diabetes Care. 2003;26(3):942-3. PubMed PMID: 12610062.

122. Meigs JB, Cagliero E, Dubey A, Murphy-Sheehy P, Gildesgame C, Chueh H, et al. A controlled trial of web-based diabetes disease management: the MGH diabetes primary care improvement project. Diabetes Care. 2003;26(3):750-7.

123. Montori VM, Dinneen SF, Gorman CA, Zimmerman BR, Rizza RA, Bjornsen SS, et al. The impact of planned care and a diabetes electronic management system on community-based diabetes care: the Mayo Health System Diabetes Translation Project. Diabetes Care. 2002;25(11):1952-7. PubMed PMID: 12401738.

124. Tierney WM, Overhage JM, Murray MD, Harris LE, Zhou XH, Eckert GJ, et al. Effects of computerized guidelines for managing heart disease in primary care. J Gen Intern Med. 2003;18(12):967-76. PubMed PMID: 14687254.

125. Murray MD, Harris LE, Overhage JM, Zhou XH, Eckert GJ, Smith FE, et al. Failure of computerized treatment suggestions to improve health outcomes of outpatients with uncomplicated hypertension: results of a randomized controlled trial. Pharmacotherapy. 2004;24(3):324-37. PubMed PMID: 15040645.

126. Lobach DS, G.D; Bright, T.J.; Wong, A.; Dhurjati, R.; Bristow, E.; Bastian, L.; Coeytaux, R.; Samsa, G.;Hasselblad, V.; Williams, J.W..; Wing, L.; Musty, M.l; Kendrick, A.S. Enabling Health Care Decisionmaking Through Clinical Decision Support and Knowledge Management. Evidence Report No. 203. Rockville, MD: AHRQ Publication No. 12-E001-EF. ; April 2012.
