# Supplementary material for: Effect of Clinical Decision Support on Cardiovascular Risk Among Adults With Bipolar Disorder, Schizoaffective Disorder, or Schizophrenia: A Cluster Randomized Clinical Trial
Source: JAMA Netw Open. 2022 Mar 7;5(3):e220202. doi: 10.1001/jamanetworkopen.2022.0202 (PMC8902652; doi:10.1001/jamanetworkopen.2022.0202)
Supplement: Supplement 2. — eTable 1. Serious Mental Illness (SMI) Diagnostic Codes eTable 2. Potentially Obesogenic Medications for Serious Mental Illness (SMI) eTable 3. Intervention Dose, ie, the Number and Percent of Visits (Index and Post-Index) at Which the CDS Was Printed by System and Treatment Group eTable 4. Rate Ratios Estimating Rates of Change in Total Modifiable CV Risk From Index to 12 Months Post-Index Among Patients in Intervention Clinics by Print Rate Subgroups eTable 5. Emergency Department Visit Rates by Health System and Treatment Group in the Year Prior to the First Visit (Pre) and Time Between First Visit and 9/19/2018 (Post) eTable 6. Inpatient Stay Rates by Health System and Treatment Group in the Year Prior to the First Visit (Pre) and Time Between First Visit and 9/19/2018 (Post) eTable 7. Suicide Attempt Rates by Health System and Treatment Group in the Year Prior to the First Visit (Pre) and Time Between First Visit and 9/19/2018 (Post) [file jamanetwopen-e220202-s002.pdf]

Supplementary Online Content

Rossom RC, Crain AL, O'Connor PJ, et al. Effect of clinical decision support on cardiovascular risk among adults with bipolar disorder, schizoaffective disorder, or schizophrenia: a cluster randomized clinical trial. *JAMA Netw Open.* 2022;5(3):e220202. doi:10.1001/jamanetworkopen.2022.0202

eTable 1. Serious Mental Illness (SMI) Diagnostic Codes
eTable 2. Potentially Obesogenic Medications for Serious Mental Illness (SMI)
eTable 3. Intervention Dose, ie, the Number and Percent of Visits (Index and Post-Index) at Which the CDS Was Printed by System and Treatment Group
eTable 4. Rate Ratios Estimating Rates of Change in Total Modifiable CV Risk From Index to 12 Months Post-Index Among Patients in Intervention Clinics by Print Rate Subgroups
eTable 5. Emergency Department Visit Rates by Health System and Treatment Group in the Year Prior to the First Visit (Pre) and Time Between First Visit and 9/19/2018 (Post)
eTable 6. Inpatient Stay Rates by Health System and Treatment Group in the Year Prior to the First Visit (Pre) and Time Between First Visit and 9/19/2018 (Post)
eTable 7. Suicide Attempt Rates by Health System and Treatment Group in the Year Prior to the First Visit (Pre) and Time Between First Visit and 9/19/2018 (Post)

This supplementary material has been provided by the authors to give readers additional information about their work.

**eTable 1. Serious Mental Illness (SMI) Diagnostic Codes**		
	***ICD-9* codes**	***ICD-10* codes**
**Bipolar disorder**	296.00-296.89, 301.11	F30.1-F31.9
**Schizophrenia**	295.0-295.6, 295.8-295.9, 297.1, 297.3, 298.8, 298.9, 301.22	F20.0-F24, F28-F29
**Schizoaffective disorder**	295.6	F25.0-F25.9

© 2022 Rossom RC et al. *JAMA Network Open.*

eTable 2. Potentially Obesogenic Medications for Serious Mental Illness (SMI)

Amitriptyline	Clozapine	Mirtazapine	Phenelzine	Thioridazine
Asenapine	Desipramine	Nortriptyline	Quetiapine	Tranylcypromine
Chlorpromazine	Doxepin	Olanzapine	Risperidone	
Clomipramine	Imipramine	Paroxetine	Selegiline	

Identified in consultation with clinic leadership and informed by a literature search. Mood-stabilizing medications were not included, as study team members and clinic leaders were unable to identify suitable non-obesogenic alternatives.

© 2022 Rossom RC et al. *JAMA Network Open.*

eTable 3. Intervention Dose, ie, the Number and Percent of Visits (Index and Post-Index) at Which the CDS Was Printed by System and Treatment Group								
	**All**		**Site A**		**Site B**		**Site C**	
	**Control**	**CDS**	**Control**	**CDS**	**Control**	**CDS**	**Control**	**CDS**
**n**	4387	4550	2120	1807	1486	1818	781	925
**visits, n**								
**M**	6.8	6.8	7.4	7.2	6.7	7.2	5.0	5.2
**SD**	5.5	5.6	6.1	6.2	5.1	5.7	3.4	3.7
**25th percentile**	3	3	3	3	3	3	3	3
**Median**	5	5	6	5	5	6	4	4
**75th percentile**	9	9	9	9	8	9	6	6
**Visits for which CDS printed, n**								
**M**	0.5	4.0	0.8	4.3	0.2	4.1	0.1	3.1
**SD**	1.5	3.3	2.0	3.7	0.9	3.3	0.5	2.3
**25th percentile**	0	2	0	2	0	2	0	2
**Median**	0	3	0	3	0	3	0	3
**75th percentile**	0	5	1	6	0	6	0	4
**CDS printed, % of visits**								
**M**	5.7	61.1	9.3	62.1	2.5	59.5	2.0	62.2
**SD**	15.3	27.8	19.0	28.7	9.5	26.4	9.6	28.3
**25th percentile**	0	45.5	0	45.5	0	44.4	0	50.0
**Median**	0	63.6	0	66.7	0	61.1	0	66.7
**75th percentile**	0	80.0	9.1	83.3	0	76.9	0	80.0
**CDS printed, % of visits**								
**0%**	82.6	5.9	71.9	6.5	91.5	5.3	94.6	5.9
**>0 - <50%**	12.6	20.5	20.0	19.9	7.1	22.2	3.1	18.3
**50%- <100%**	4.6	56.0	7.7	54.1	1.3	58.7	2.3	54.3
**100%**	0.2	17.6	0.4	19.4	0	13.8	0	21.5

© 2022 Rossom RC et al. *JAMA Network Open.*

eTable 4. Rate Ratios Estimating Rates of Change in Total Modifiable CV Risk From Index to 12 Months Post-Index Among Patients in Intervention Clinics by Print Rate Subgroups

	**ALL**	**Site A**	**Site B**	**Site C**
	**RR (95% CI)**	**RR (95% CI)**	**RR (95% CI)**	**RR (95% CI)**
**Print rate**				
**0%**	0.99 (0.91-1.07)	0.93 (0.83-1.03)	1.24 (1.06-1.45)	0.83 (0.61-1.11)
**>0 - <50%**	0.99 (0.96-1.02)	1.00 (0.96-1.04)	0.98 (0.94-1.02)	1.00 (0.91-1.09)
**50%- <100%**	0.99 (0.98-1.01)	0.99 (0.96-1.01)	1.01 (0.98-1.04)	0.97 (0.92-1.04)
**100%**	1.01 (0.96-1.06)	1.01 (0.95-1.08)	0.99 (0.91-1.08)	1.03 (0.90-1.19)

© 2022 Rossom RC et al. *JAMA Network Open.*

eTable 5. Emergency Department Visit Rates by Health System and Treatment Group in the Year Prior to the First Visit (Pre) and Time Between First Visit and 9/19/2018 (Post)

		Site A			Site B			Site C		
		**Control**	**CDS**	**All**	**Control**	**CDS**	**All**	**Control**	**CDS**	**All**
**n patients**		2120	1807	3927	1486	1818	3304	781	925	1706
**pt years**	**M**	2.07	2.07	2.07	1.42	1.42	1.42	1.31	1.32	1.32
**follow-up**	**Mdn**	2.21	2.21	2.21	1.48	1.48	1.48	1.35	1.36	1.36
**n ED visits**	**pre**	2053	1037	3090	1933	1776	3709	248	464	712
	**post**	3873	2218	6091	2601	2316	4917	389	839	1228
**n per patient**	**pre**	0.97	0.57	0.79	1.30	0.98	1.12	0.32	0.50	0.42
	**post**	1.83	1.23	1.55	1.75	1.27	1.49	0.50	0.91	0.72
**n patients with 1+**	**pre**	762	395	1157	626	621	1247	125	212	337
	**post**	1008	576	1584	713	708	1421	161	309	470
**% of patients with 1+**	**pre**	35.9	21.9	29.5	42.1	34.2	37.7	16.0	22.9	19.8
	**post**	47.5	31.9	40.3	48.0	38.9	43.0	20.6	33.4	27.5
			tx, p=	0.02		tx, p=	0.42		tx, p=	0.12
			time, p=	0.02		time, p=	0.20		time, p=	0.07
			tx*time, p=	0.40		tx*time, p=	0.80		tx*time, p=	0.20
**n per patient-year**	**pre**	0.97	0.57	0.79	1.30	0.98	1.12	0.32	0.50	0.42
	**post**	0.88	0.59	0.75	1.23	0.90	1.05	0.38	0.69	0.55
			tx, p=	0.14		tx, p=	0.24		tx, p=	0.17
			time, p=	0.61		time, p=	0.16		time, p=	0.04
			tx*time, p=	0.17		tx*time, p=	0.73		tx*time, p=	0.42

© 2022 Rossom RC et al. *JAMA Network Open.*

eTable 6. Inpatient Stay Rates by Health System and Treatment Group in the Year Prior to the First Visit (Pre) and Time Between First Visit and 9/19/2018 (Post)

		Site A			Site B			Site C		
		Control	CDS	All	Control	CDS	All	Control	CDS	All
n patients		2120	1807	3927	1486	1818	3304	781	925	1706
pt years	M	2.07	2.07	2.07	1.42	1.42	1.42	1.31	1.32	1.32
follow-up	Mdn	2.21	2.21	2.21	1.48	1.48	1.48	1.35	1.36	1.36
n IP stays	pre	420	247	667	357	408	765	84	153	237
	post	729	509	1238	593	600	1193	93	222	315
n per patient	pre	0.20	0.14	0.17	0.24	0.22	0.23	0.11	0.17	0.14
	post	0.34	0.28	0.32	0.40	0.33	0.36	0.12	0.24	0.19
n patients with 1+	pre	279	167	446	221	235	456	53	100	153
	post	383	259	642	287	313	600	64	130	194
% of patients with 1+	pre	13.2	9.2	11.4	14.9	12.9	13.8	6.8	10.8	9.0
	post	18.1	14.3	16.3	19.3	17.2	18.2	8.2	14.1	11.4
			tx, p=	0.09		tx, p=	0.40		tx, p=	0.11
			time, p=	0.0002		time, p=	0.84		time, p=	0.88
			tx*time, p=	0.31		tx*time, p=	0.85		tx*time, p=	0.69
n per patient-year	pre	0.20	0.14	0.17	0.24	0.22	0.23	0.11	0.17	0.14
	post	0.17	0.14	0.15	0.28	0.23	0.25	0.09	0.18	0.14
			tx, p=	0.31		tx, p=	0.59		tx, p=	0.09
			time, p=	0.28		time, p=	0.06		time, p=	0.71
			tx*time, p=	0.16		tx*time, p=	0.16		tx*time, p=	0.25

© 2022 Rossom RC et al. *JAMA Network Open.*

eTable 7. Suicide Attempt Rates by Health System and Treatment Group in the Year Prior to the First Visit (Pre) and Time Between First Visit and 9/19/2018 (Post)

		Site A			Site B			Site C		
		**Control**	**CDS**	**All**	**Control**	**CDS**	**All**	**Control**	**CDS**	**All**
**n patients**		2120	1807	3927	1486	1818	3304	781	925	1706
**pt years**	**M**	2.07	2.07	2.07	1.42	1.42	1.42	1.31	1.32	1.32
**follow-up**	**Mdn**	2.21	2.21	2.21	1.48	1.48	1.48	1.35	1.36	1.36
**n suicide attempts**	**pre**	57	42	99	64	66	130	3	5	8
	**post**	93	60	153	67	48	115	10	18	28
**n per patient**	**pre**	0.027	0.023	0.025	0.043	0.036	0.039	0.004	0.005	0.004
	**post**	0.044	0.033	0.039	0.045	0.026	0.035	0.013	0.019	0.016
**n patients with 1+**	**pre**	49	33	82	33	30	63	3	5	8
**suicide attempt**	**post**	69	48	117	41	27	68	8	5	13
**% of patients with 1+**	**pre**	2.3	1.8	2.1	2.2	1.7	1.9	0.4	0.5	0.5
**suicide attempts**	**post**	3.3	2.7	3.0	2.8	1.5	2.1	1.0	0.5	0.8
			tx, p=	0.21		tx, p=	0.009			
			time, p=	0.02		time, p=	0.10			
			tx*time, p=	0.94		tx*time, p=	0.36			
**n per patient-year**	**pre**	0.027	0.023	0.025	0.043	0.036	0.039	.004	.005	.005
	**post**	0.021	**0.016**	0.019	**0.032**	**0.019**	0.025	**.010**	**.015**	.012
			tx, p=	0.44		tx, p=	0.22			
			time, p=	0.05		time, p=	0.09			
			tx*time, p=	0.61		tx*time, p=	0.41			

© 2022 Rossom RC et al. *JAMA Network Open.*
