# Supplementary material for: Effect of Clinical Decision Support on Cardiovascular Risk Among Adults With Bipolar Disorder, Schizoaffective Disorder, or Schizophrenia: A Cluster Randomized Clinical Trial
Source: JAMA Netw Open. 2022 Mar 7;5(3):e220202. doi: 10.1001/jamanetworkopen.2022.0202 (PMC8902652; doi:10.1001/jamanetworkopen.2022.0202)
Supplement: Supplement 3. — Data Sharing Statement [file jamanetwopen-e220202-s003.pdf]

Data Sharing Statement

Rossom. Effect of Clinical Decision Support on Cardiovascular Risk Among Adults With Bipolar Disorder, Schizoaffective Disorder, or Schizophrenia. *JAMA Netw Open*. Published March 07, 2022. doi:10.1001/jamanetworkopen.2022.0202

Data
Data available: No

Additional Information
Explanation for why data not available: Individual-level data are not available for data sharing. Sharing of summary data will be considered on request.
